# Health sequelae of human cryptosporidiosis in industrialised countries: a systematic review

**DOI:** 10.1186/s13071-020-04308-7

**Published:** 2020-09-04

**Authors:** Bethan L. Carter, Rachel M. Chalmers, Angharad P. Davies

**Affiliations:** 1 Swansea University Medical School, Swansea University, Singleton Park, Swansea, UK; 2 Cryptosporidium Reference Unit, Public Health Wales Microbiology, Singleton Hospital, Sketty Lane, Swansea, Wales UK

**Keywords:** Cryptosporidiosis, Sequelae, *Cryptosporidium hominis*, *Cryptosporidium parvum*

## Abstract

**Background:**

*Cryptosporidium* is a protozoan parasite which is a common cause of gastroenteritis worldwide. In developing countries, it is one of the most important causes of moderate to severe diarrhoea in young children; in industrialised countries it is a cause of outbreaks of gastroenteritis associated with drinking water, swimming pools and other environmental sources and a particular concern in certain immunocompromised patient groups, where it can cause severe disease. However, over recent years, longer-term sequelae of infection have been recognised and a number of studies have been published on this topic. The purpose of this systematic review was to examine the literature in order to better understand the medium- to long-term impact of cryptosporidiosis.

**Methods:**

This was a systematic review of studies in PubMed, ProQuest and Web of Science databases, with no limitations on publication year or language. Studies from any country were included in qualitative synthesis, but only those in industrialised countries were included in quantitative analysis.

**Results:**

Fifteen studies were identified for qualitative analysis which included 3670 *Cryptosporidium* cases; eight studies conducted in Europe between 2004–2019 were suitable for quantitative analysis, including five case-control studies. The most common reported long-term sequelae were diarrhoea (25%), abdominal pain (25%), nausea (24%), fatigue (24%) and headache (21%). Overall, long-term sequelae were more prevalent following infection with *Cryptosporidium hominis*, with only weight loss and blood in stool being more prevalent following infection with *Cryptosporidium parvum*. Analysis of the case-control studies found that individuals were 6 times more likely to report chronic diarrhoea and weight loss up to 28 months after a *Cryptosporidium* infection than were controls. Long-term abdominal pain, loss of appetite, fatigue, vomiting, joint pain, headache and eye pain were also between 2–3 times more likely following a *Cryptosporidium* infection.

**Conclusions:**

This is the first systematic review of the long-term sequelae of cryptosporidiosis. A better understanding of long-term outcomes of cryptosporidiosis is valuable to inform the expectations of clinicians and their patients, and public health policy-makers regarding the control and prevention of this infection.

*Systematic review registration* PROSPERO Registration number CRD42019141311
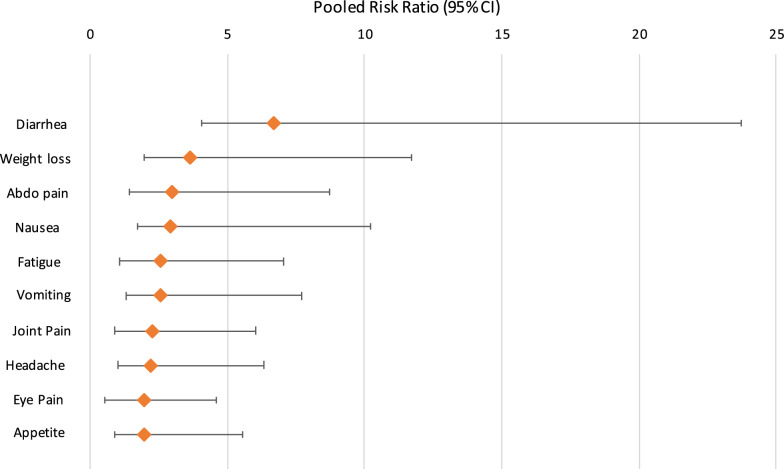

## Background

Cryptosporidiosis is a clinical disease, typically affecting the intestinal tract of humans and animals who have ingested the protozoan parasite *Cryptosporidium* in its oocyst (infective) stage [1]. Transmission of *Cryptosporidium* occurs predominantly *via* the faecal-oral route, or through consumption of contaminated food or water and therefore the prevalence of human *Cryptosporidium* infections is higher in low-resource settings [2]. However, *Cryptosporidium* infections are not infrequent in industrialized countries [3], with large outbreaks being reported in Sweden [4], the USA [5, 6] and the UK [7] following contamination of public water supplies.

While asymptomatic carriage is possible [8, 9], human cryptosporidiosis typically presents as an acute, gastroenteritis-like illness characterized by profuse, watery diarrhoea, frequently accompanied by abdominal pain/cramps, vomiting and weight loss, as well as more non-specific symptoms such as fatigue, low-grade fever, nausea and muscle weakness [10]. In immunocompetent hosts, cryptosporidiosis is generally self-limiting; however, disease severity can be influenced by host factors, such as age, immune status and nutritional status, as well as pathogen factors e.g. *Cryptosporidium* species and subtype [11].

Alongside ongoing interest in the acute symptomology of human cryptosporidiosis, there is also growing evidence to suggest that, rather like some bacterial causes of gastroenteritis and giardiasis [12–14], *Cryptosporidium* infection may have longer-term health consequences. Seven studies [15–21], with follow-up periods ranging from 2 months to 3 years, have investigated numerous potential post-*Cryptosporidium* infection sequelae including diarrhoea, abdominal pain, vomiting, loss of appetite, irritable bowel syndrome (IBS) [21], joint pain and fatigue, while case reports document incidences of reactive arthritis [22–24], Reiter’s syndrome [25], acute pancreatitis [26, 27] and haemolytic uremic syndrome [28], in the context of *Cryptosporidium* infection. There is also some emerging evidence, recently reviewed, of a possible association between cryptosporidiosis and cancer [29].

Due to resource limitations, public health professionals currently face the challenge of identifying and prioritising specific infectious diseases whose quantified burden of disease estimates justify the allocation of interventions and funding for research [30]. The Global Enteric Multicentre Study [31] identified *Cryptosporidium* as the second most common cause of moderate-to-severe diarrhoea (MSD; defined as diarrhoeal disease with presence of the suggestive features of sunken eyes, wrinkled skin, hospitalization, receipt of intravenous hydration, or dysentery) in children less than 2 years-old within sub-Saharan Africa and south Asia, while in 2016, the European Network for Foodborne Parasites (Euro-FBP) ranked *Cryptosporidium* spp. as the second highest priority foodborne parasite in northern and western Europe, and the eighth highest priority in eastern and south-western Europe [30]. However, actual burden of disease estimates for *Cryptosporidium* still vary widely [11] and it remains difficult to quantify the true burden of cryptosporidiosis, as current estimates only account for the morbidity and mortality associated with the acute illness, while the potential contributions of long-term manifestations are not included [32, 33]. A recent study from the Netherlands [2] found that long-term manifestations contributed nearly 10% of the total Disability-Adjusted Life Years (DALYs) and costs when included in burden of disease models for *Cryptosporidium*, suggesting a higher public heath burden and cost than previously estimated.

Accurate estimations of the burden of disease associated with *Cryptosporidium* will inform decisions regarding the allocation of diagnostic, surveillance and interventional measures to prevent and control *Cryptosporidium* infections. Due to the potential morbidity and mortality associated with long-term sequelae of human cryptosporidiosis, an accurate estimation of the proportion of cases that develop such sequelae is needed to quantify true burden of disease estimates for *Cryptosporidium*.

The objectives of this review were: (i) estimate the proportion of people that self-report health sequelae post-*Cryptosporidium* infection; (ii) estimate the risk of specific sequelae following *Cryptosporidium* infection; and (iii) explore potential risk factors associated with developing sequelae following *Cryptosporidium* infection in industrialised countries.

## Methods

### Search strategy

We searched for studies in PubMed, ProQuest and Web of Science databases, with no limitations on publication year or language. The reference lists from relevant papers identified during our electronic searches were also reviewed for additional relevant papers which may warrant inclusion in our review. Search terms were initially developed and piloted using PubMed and, to ensure consistency, the same search terms were used when searching ProQuest and Web of Science databases. Databases were searched using the following keywords: Cryptosporid*, Complications, Sequel*, Post-infecti*, Long term and Chronic. The full electronic search strategies are documented in Additional file 1: Table S1. The review was registered with PROSPERO, registration number CRD42019141311.

#### Selection of studies

All citations identified using the final search strategies were exported to Mendeley® reference managing software for organisation and removal of duplicates. The titles and abstracts of the remaining articles were screened for relevance by one reviewer (BC), after which, the remaining articles were independently screened by two reviewers (BC and APD) to ensure consistent application of the pre-determined inclusion/exclusion criteria (Additional file 1: Table S1).

Studies from any country were included in qualitative synthesis, but only those in industrialised countries were included in quantitative analysis. An industrialised country was defined using Organisation for Economic Co-operation & Development (OECD) membership.

Final inclusion of studies was decided by consensus, with any conflicts being reviewed by a third reviewer (RMC). The full text was retrieved and reviewed for articles where the title and abstract had been deemed relevant by reviewers.

#### Data extraction

Data were extracted from eligible studies and collated in a Microsoft Word document. We recorded post-*Cryptosporidium* infection health sequelae data as reported in the individual papers (e.g. prevalence, cumulative incidence, etc.). Relative risks or odds ratios were recorded where data were available. We also extracted the following study characteristics from each paper if available: name of authors, year of publication, study location/setting, study design, year(s) of study, study duration and duration of follow-up, number of included study participants, participation rate, study population demographics (including age and gender distributions), *Cryptosporidium* species data, the diagnostic method to ascertain *Cryptosporidium* infection and the types of sequelae reported. Additionally, where available, data on the incidence/prevalence of post-infectious IBS following *Cryptosporidium* infection and the IBS diagnostic criterion applied were collected.

#### Quality assessment

The methodological quality of the included studies was assessed using the Newcastle-Ottawa Scale (NOS) for nonrandomised studies [34]. NOS was used to score studies using three domains: (i) the selection of the study groups; (ii) the comparability of the groups; and (iii) the determination of either the exposure or outcome of interest in case-control or cohort studies, respectively. Scores ranged between 5–8 (Additional file 1: Table S2).

### Statistical analysis

The proportion of *Cryptosporidium* cases that developed specific sequelae was calculated by dividing the number of individuals developing a sequela by the total number of *Cryptosporidium* cases. Where data were available from two or more appropriate studies, we used a random-effects meta-analysis model to obtain pooled estimates of prevalence for the outcomes of interest (i.e. sequelae) across the eligible studies. For this analysis, a study could be included more than once if sequelae data were reported longitudinally at different time periods. Data analyses were performed using Meta XI [35].

### Assessment of heterogeneity and reporting biases

Forest plots and the I^2^ statistic were used to assess heterogeneity between the studies. Values of 0–40%, 30–60%, 50–90% and 75–100% were interpreted as; might not be important, may represent moderate heterogeneity, may represent substantial heterogeneity and considerable heterogeneity, respectively [36]. Funnel plots were used to assess for publication bias and small-study effects.

Stratified analysis was performed for the following subgroups; time (less than 6 months post-infection and more than 6 months post-infection) and *Cryptosporidium* sp. (e.g. *C. parvum vs C. hominis*).

## Results

### Data synthesis

**Fig. 1:**
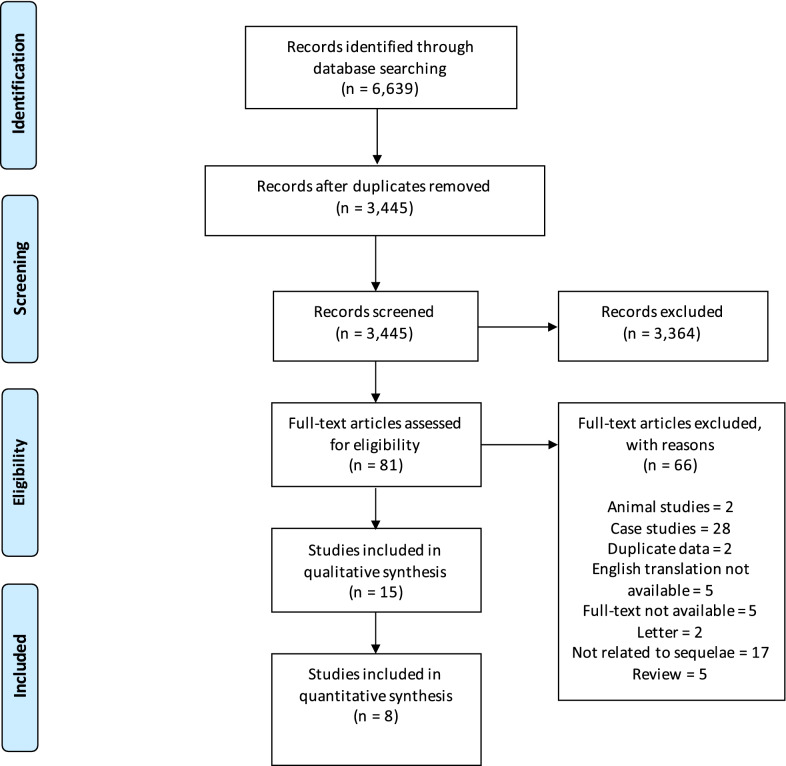
PRISMA flowchart

The number of papers identified, included and excluded is presented according to the requirements of the PRISMA statement [37] in Fig. 1. Fifteen studies were identified for qualitative synthesis and eight of these were identified as being set in industrialised countries and of sufficient quality for additional quantitative synthesis.

**Fig. 2:**
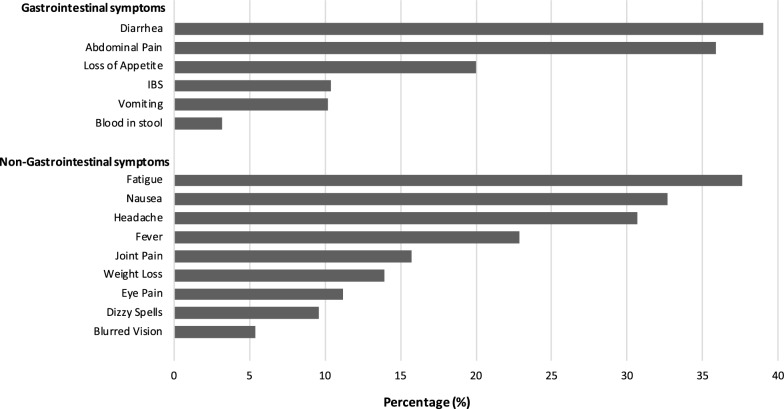
Reported sequelae up to 36 months post-*Cryptosporidium* infection

**Fig. 3:**
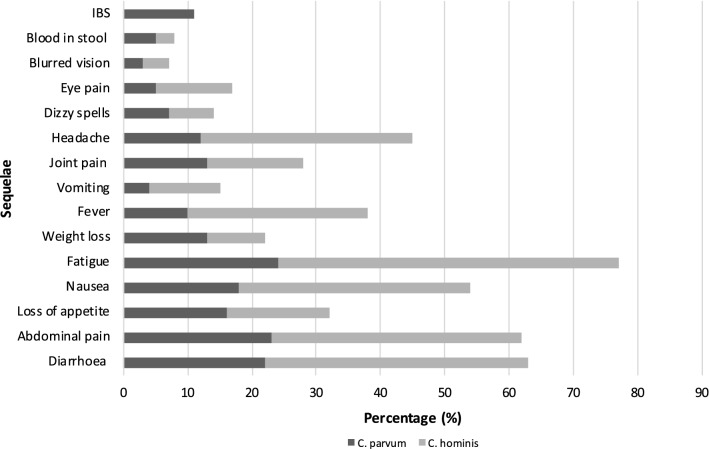
Reported sequelae up to 36 months post-*Cryptosporidium* infection by species (%)

The qualitative synthesis is shown in Table 1. Quantitative synthesis results are shown in Tables 2, 3 and 4 and Figs. 2 and 3.

**Table 1:** Fifteen studies included in the qualitative synthesis

References/location	Study setting, design and duration of follow-up	*Cryptosporidium* spp.	Sample size	Range of age/sex	Main findings
Agnew et al. [38] Fortaleza, Brazil	Urban slum Nested case-control study of a cohort of young children Cases (diagnosed cryptosporidiosis): 453 ± 49 (15–1167) days Controls: 436 ± 53 (0–1165) days	Unidentified	43 cases; 43 controls	Age of cases (months): 11 ± 0.9 (range: 3–26) Age of controls (months): 11 ± 0.9 (4–27) Cases: 63% boys Controls: 40% boys	Children who had an episode of symptomatic *Cryptosporidium* infection had a significantly increased diarrhoeal disease burden (days of diarrhoea/child-year) compared with that for controls both before (39.3 ± 7 *vs* 21.3 ± 5 days, respectively; *P* < 0.04) and after (46.1 ± 9 *vs* 13.9 ± 5 days, respectively; *P* < 0.04) the diagnosis of *Cryptosporidium* infection In the post-*Cryptosporidium* period, case-children who were < 1 year of age had significantly more episodes of diarrhoea than their controls and significantly more episodes of diarrhoea than in their pre-*Cryptosporidium* period (data not shown; *P* ≤ 0.001 and *P* < 0.05, respectively) Before *Cryptosporidium* infection, 8 case-children, who were ≤ 1 year-old and had no diarrhoeal illnesses, had height-for-age *Z*-scores identical to matched controls. However, after *Cryptosporidium* infection, these case-children had significant decline in height-for-age *Z*-scores which were not seen in the matched controls (*P* < 0.005 for pre-infection *vs* post-infection case-children) It is not known whether the increase in post-*Cryptosporidium* diarrhoeal disease burden was due solely to the impact of infection with *Cryptosporidium*, or if a similar phenomenon would also be seen with other serious enteric infections (e.g. rotavirus or enteroaggregative *Escherichia coli*)
Ajjampur et al. [39] Vellore, South India	Semi-urban slum Prospective birth cohort study 3 years	Unidentified	40/116 children who consented to take part in the study were identified as having had cryptosporidial diarrhoea, 66 of them had giardial diarrhoea and 22 had both 32 with no documented episodes of cryptosporidial or giardial diarrhoea were also recruited	Mean (± SD) age of the children during assessment was 3.51 ± 0.38 years Median (IQR) for age at the first documented cryptosporidial episodes were 1.29 (0.81–2.05) years 55.2% males	Children with cryptosporidial diarrhoea had a mean (SD) social quotient (SQ) of 118.70 (35.01) (*P* = 0.714) Children with cryptosporidial diarrhoea did not have significantly lower IQ scores than those without a past history of cryptosporidial diarrhoea (mean IQ 100.12, SD 17.28) In the univariate analysis, a past history of any protozoan diarrhoea, either giardial or cryptosporidial, was not a significant predictor of stunting or being underweight Cryptosporidial diarrhoea was not associated with poor IQ, SQ or physical growth
Berkman et al. [40] Lima, Peru	Periurban shanty town Prospective birth cohort Follow-up birth to 2 years with cognitive function at 9 years	Unidentified	Cognitive assessment completed in 143 children 77 (54%) had at least one episode of *Cryptosporidium* infection	Follow-up birth to 2 years with cognitive function at 9 years. Estimated median age at onset of first *Cryptosporidium* infection was 16.1 months 76 (53%) males	No association between *Cryptosporidium* infection and cognitive test scores according to the number of episodes, incidence and prevalence, and symptomatic infections
*Carter et al. [21] UK (Wales)	Sporadic community cases Prospective case cohort study 12 months	*C. parvum* (*n* = 121) *C. hominis* (*n* = 79) *C. parvum* and *C. hominis* (*n* = 2) Other species (*n* = 3)	515 eligible; 205 participated	42 (20%): 6 months-4 years 63 (31%): 5–17 years 100 (49%): 18 years or over 60.6% female at baseline 58.2% female at 3 months 66.3% female at 12 months	12 months follow-up: over a third of cases reported persistent abdominal pain and diarrhoea, 28% reported joint pain and 26% reported fatigue At both 3 and 12 months, the proportion reporting fatigue and abdominal pain after *C. hominis* infection was statistically significantly greater than after *C. parvum* Overall, 10% of cases had sufficient symptoms to meet IBS diagnostic criteria. A further 27% met all criteria except 6 months’ duration and another 23% had several features of IBS but did not fulfil strict Rome III criteria. There was no significant difference between *C. parvum* and *C. hominis* infection with regard to PI-IBS
Delahoy et al. [41] Kenya	Rural community Prospective, age-stratified, health facility-based matched case-control study of children with MSD ~ 60 days (acceptable range 50–90 days)	Unidentified	Among the 1778 MSD case children enrolled, *Cryptosporidium* was identified in 195 cases (11.0%)	46%: 0–11 months 27%: 12–23 months 25%: 24–59 months 56% male	At follow-up, *Cryptosporidium*- positive cases had increased odds of being stunted (adjusted odds ratio, aOR: 1.65, 95% CI: 1.06–2.57), underweight (aOR: 2.08, 95% CI: 1.34–3.22), or wasted (aOR: 2.04, 95% CI: 1.21–3.43), and had significantly larger negative changes in height- and weight-for-age z-scores from enrollment
Guerrant et al. [42] Fortaleza, Brazil	Urban slum Prospective cohort study 6–9 years	Unidentified	26 children; 9 *Cryptosporidium* infections (6 with diarrhoea, 3 without diarrhoea)	26 children (12 boys and 14 girls) Age range: 6.5–9 years	*Cryptosporidium* infections (seen in 9/26 children) in the first 2 years of life were correlated with a 2-fold increase in episodes of diarrhoea at 0–2 years of age (*P* = 0.017, by 2-sample t-test) Fitness scores in children with early childhood *Cryptosporidium* were 10% lower than in controls (9.0 *vs* 10.0; *P* = 0.008, by 2-sample t-test) Adjusting for *Cryptosporidium* removed both the significance of the correlation between diarrhoea and fitness and between *Cryptosporidium* and fitness
*Hunter et al. [15] UK (Northwest of England and Wales)	Sporadic community cases and controls Case-control study 2 months	*C. parvum* (*n* = 50) *C. hominis* (*n* = 61) Unidentified (*n* = 124)	235 case patients; 232 control subjects	Age range: 0–89 years Control subjects were significantly older than case patients (*χ*^2^ = 8.574, *P* = 0.0034) 49% of case patients and 46% of control subjects were male	40% of case patients reported recurrence of intestinal symptoms after resolution of the acute stage of illness Reports of joint pain (odds ratio, OR: 2.8), eye pains (OR: 2.44), recurrent headache (OR: 2.10), dizzy spells (OR: 1.69), and fatigue (OR: 3.0) were significantly more common in case patients than in control subjects, but only in people who had experienced *C. hominis* infection
*Igloi et al. [15] Netherlands	Sporadic community cases and controls Case-crossover and cryptosporidiosis case control study 4 months	*C. parvum* (*n* = 216) *C. hominis* (*n* = 92)	308 cases	Median age: 26 years (range: 1–80) 58% were female	Compared to before illness, cases were significantly more likely to report dizziness (OR: 2.25), headache (OR: 2.15), fatigue (OR: 2.04), weight loss (OR: 1.82), diarrhoea (OR: 1.50), abdominal pain (OR: 1.38) or joint pain (OR: 1.84). However, symptoms of joint pain and headache occurred among cases after illness at a rate that was not significantly different from that observed in the general population There were no significant differences in post-infection symptom occurrence between *C. hominis* and *C. parvum*
*Insulander et al. [19] Stockholm County, Sweden	Sporadic community cases Prospective cryptosporidiosis case cohort study 25–36 months	*C. parvum* (n = 111) *C. hominis* (n = 65) Other species (*n* = 17)	271 cases	Median age: 32 years (range: 1–73 years) 126 male and 145 female	After 25–36 months follow-up: 15% reported intermittent diarrhoea (8/53), 9% reported abdominal pain (5/53), 8% reported myalgia/arthralgia (4/53), 4% reported fatigue (2/53) There was no difference in frequency of persisting symptoms between patients infected with *C. parvum* or *C. hominis*
Korpe et al. [43] Bangladesh	Peri-urban slum Prospective birth cohort study 2 years	*C. hominis* (*n* = 220) *C. parvum* (*n* = 8) *C. parvum* and *C. hominis* (*n* = 5) Other species (*n* = 5) Unidentified (*n* = 154)	392 children	Birth to 24 months of age 55% male	Children with *Cryptosporidium* spp. infection had a greater than 2-fold increased risk of severe stunting at age two compared to uninfected children (OR: 2.69, 95% CI 1.17–6.15, *P* = 0.019) independent of sex, income, maternal body-mass index, maternal education and weight for age adjusted *z-*score (WAZ) at birth
*Lilja et al. [20] Ostersund, Sweden	Outbreak cohort and controls Case-control study 28 months	*C. hominis*	215 cases; 344 non-cases	Median age of cases: 41 (range: 3–79) years Median age of non-cases: 56 (range: 3–95) years 57% of cases and 55% of controls were women	48% of cases reported symptoms at follow-up, most commonly headache, fatigue, abdominal pain, and nausea Compared to non-cases, the cases were more likely to report watery diarrhoea, abdominal pain, stiff joints, joint pain, joint discomfort, fatigue, nausea, and headache at follow-up after adjusting for age and sex The likelihood of cases reporting symptoms at follow-up differed between age groups: joint pain (OR: 13.2, 95% CI: 2.8–61.9) and nausea (OR: 2.7, 95% CI: 1.2–6.0) were associated only with the 16–40-year age group; diarrhoea (OR: 3.9, 95% CI: 1.1–14.3) was associated only with the > 65-year age group; and headache (OR: 4.0, 95% CI: 1.3–13.1) was associated only with the 6–15-year-old age group
Phillips et al. [44] London, UK	Sporadic urban community and traveller community cases Retrospective cohort Variable	Unidentified	123 children	Not specified	50% of children excreting only *Cryptosporidium* had diarrhoea lasting over 21 days; in 8% of cases diarrhoea continued for over 6 months. 23% of cases had weight below the third centile and a further 9% had failure to thrive. Most cases (63%) of chronic diarrhoea occurred in the first two years of life. A mild to moderate enteropathy was present in all 9 children undergoing a small intestinal biopsy and 7 showed the presence of *Cryptosporidium* adhering to villous epithelium. All patients eventually recovered spontaneously Although a greater proportion of patients with mixed infections had weight below the 3rd percentile (8/21) this was not significantly different to those with *Cryptosporidium* alone (11/61)
*Rehn et al. [17] Ostersund and Skelleftea, Sweden	Community outbreak cases and controls Case-control study 11 months	*C. hominis*	Östersund: 872 (310 cases) Skellefteå: 743 (149 cases)	Östersund: Median age of cases: 32 years (range: 1–93) Skellefteå: Median age of cases: 34 years (range: 2–92) Östersund: 310 (38%) cases, 138 (45 %) were male Skellefteå study: 149 (22%) cases, 73 (49 %) were	Outbreak cases were more likely to report diarrhoea (Östersund OR: 3.3, 95% CI: 2.0–5.3. Skellefteå OR: 3.6, 95% CI: 2.0–6.6), watery diarrhoea (Östersund OR: 3.4, 95% CI: 1.9–6.3. Skellefteå OR: 2.8, 95% CI: 1.5–5.1) abdominal pain (Östersund OR: 2.1, 95% CI: 1.4–3.3, Skellefteå OR: 2.7, 95% CI: 1.5–4.6) and joint pain (Östersund OR: 2.0, 95% CI: 1.2–3.3, Skellefteå OR: 2.0, 95% CI: 1.1–3.6) at follow-up compared to non-cases
*Stiff et al. [18] UK (mainly northern England)	Community outbreak cases Prospective cohort study 12 months	*C. parvum*	197 invited; 54 took part	Mean age: 41.8 years 14 males and 40 females	12 months follow up: participants self-reported weight loss (31%), abdominal pain (38%), diarrhoea (33%), eye pain (9 %), joint pain (33 %), fatigue (22 %) and symptoms consistent with irritable bowel syndrome (IBS) (28 %). Two people were medically diagnosed with IBS
*Widerstrom et al. [4] Östersund, Sweden	Community outbreak cases and controls Case-control study 2 months	*C. hominis*	1524 eligible; 1044 (69.2%) responded	Median age: 44 years (range: 0–98 years) 481 male (46.1%) and 563 female (53.9%)	Most common symptoms among case-patients were episodes of diarrhoea > 3 times daily (89.0%), watery diarrhoea (84.3%), abdominal cramps (78.8%), fatigue (73.1%), nausea (63.9%), and headache (57.1%) Muscle or joint aches, which were reported less frequently in Östersund than in other studies The median duration of diarrhoea, the level of attack rates in different age groups, and recurrence rate of diarrhoea corresponded to findings in other outbreaks

*Note*: Eight studies which were included in the quantitative synthesis are marked by an asterisk (*)

**Table 2:** Pooled estimates for the prevalence of post-*Cryptosporidium* sequelae using a random effects model

Sequelae	No. of studies	Pooled estimate (%) (95% CI)	Cochran Q	*P*-value
Diarrhoea	13	25 (10–44)	1382.71	< 0.001
Abdominal pain	13	25 (13–39)	575.30	< 0.001
Joint pain	13	15 (12–19)	63.28	< 0.001
Fatigue	13	24 (13–37)	477.50	< 0.001
Vomiting	10	8 (5–12)	72.61	< 0.001
Headache	10	21 (12–33)	271.21	< 0.001
Eye pain	10	10 (7–14)	46.66	< 0.001
Loss of appetite	9	19 (14–24)	51.32	< 0.001
Weight loss	9	13 (7–20)	97.98	< 0.001
Nausea	8	24 (11–40)	263.50	< 0.001
Blood in stool	7	3 (2–6)	17.26	0.01
Dizzy spells	6	8 (5–12)	13.24	0.02
Fever	5	13 (4–25)	51.28	< 0.001
Blurred vision	5	6 (4–8)	5.19	0.27
IBS	3	11 (6–16)	0.11	0.95

*Note*: Studies were included more than once if outcomes were reported at more than one interval

**Table 3:** Pooled prevalence of post-*Cryptosporidium* sequelae estimated by a random effects model, according to clinical manifestation by time period post-infection

Sequelae	No. of studies	Pooled estimate (%) (95% CI)	Cochranʼs Q	*P*-value
< 6 months				
Diarrhoea	5	43 (12–77)	532.73	< 0.001
Abdominal pain	5	41 (16–68)	278.61	< 0.001
Loss of appetite	4	26 (21–32)	8.11	0.04
Nausea	3	37 (59–69)	82.86	< 0.001
Fatigue	5	39 (17–63)	227.41	< 0.001
Weight loss	4	22 (19–26)	3.20	0.36
Fever	3	15 (2–33)	37.77	< 0.001
Vomiting	5	9 (3–16)	47.83	< 0.001
Joint pain	5	18 (15–21)	5.75	0.22
Headache	5	21 (5–42)	215.84	< 0.001
Dizzy spells	4	9 (5–14)	10.81	0.01
Eye pain	5	9 (4–15)	35.88	< 0.001
Blurred vision	3	5 (3–7)	2.10	0.35
Blood in stool	3	4 (3–6)	0.00	1
> 6 months				
Diarrhoea	8	16 (13–22)	31.14	< 0.001
Abdominal pain	8	16 (9–25)	71.17	< 0.001
Loss of appetite	5	14 (10–18)	8.68	0.07
Nausea	5	18 (13–25)	19.47	< 0.001
Fatigue	8	16 (9–26)	80.85	< 0.001
Weight loss	5	6 (4–9)	6.50	0.16
Fever	2	10 (3–21)	2.57	0.11
Vomiting	5	7 (3–12)	21.53	< 0.001
Joint pain	8	14 (9–19)	45.27	< 0.001
Headache	5	22 (12–34)	52.38	< 0.001
Dizzy spells	2	6 (2–11)	0.04	0.84
Eye pain	5	12 (7–14)	8.84	0.07
Blurred vision	2	9 (4–14)	0.01	0.94
Blood in stool	4	3 (0–6)	11.78	0.01

**Table 4:** Pooled risk ratio of individual post-*Cryptosporidium* sequelae

Sequelae	No. of studies	Pooled RR (95% CI)	Cochranʼs Q	*P*-value
Diarrhoea	5	6.7 (2.63–17.03)	105.37	< 0.001
Abdominal pain	5	2.99 (1.56–5.72)	107.10	< 0.001
Loss of appetite	4	1.98 (1.48–2.63)	8.03	0.05
Nausea	3	2.89 (1.15–7.30)	56.77	< 0.001
Fatigue	5	2.56 (1.47–4.48)	108.43	< 0.001
Weight loss	4	3.65 (1.66–8.03)	22.83	< 0.001
Vomiting	5	2.56 (1.27–5.15)	32.38	< 0.001
Joint pain	5	2.26 (1.35–3.77)	34.30	< 0.001
Headache	5	2.23 (1.22–4.09)	97.31	< 0.001
Eye pain	5	1.98 (1.09–3.59)	28.90	< 0.001

*Abbreviation*: RR, risk ratio

#### Qualitative synthesis

Electronic searching returned 1251 PubMed, 2161 ProQuest and 3227 Web of Science abstracts. After removal of duplicates, screening and assessment, 15 articles were suitable for inclusion in the qualitative synthesis and the data extracted from these studies are summarized in Table 1.

The 15 shortlisted studies included 3670 *Cryptosporidium* cases. The studies comprised 8 cohort studies and 7 case-control studies. Seven studies were conducted in children, with the remaining 8 studies including both adults and children. The length of duration of follow-up ranged from 2 months to 9 years. Studies were conducted in South America (3 studies), Africa (1 study), South Asia (2 studies) and Europe (9 studies) and were all based in a community setting. The selected studies were published between 1992 and 2019. The studies investigated a range of potential sequelae; diarrhoea (3 studies), developmental delay (2 studies), stunting of growth (4 studies) and multiple gastrointestinal and non-gastrointestinal symptoms (8 studies).

#### Quantitative synthesis

Adequate information to estimate post-*Cryptosporidium* infection sequelae was available in 8 of the 15 studies [4, 15–21]. The pooled estimates for each of the sequelae are shown in Table 2. Data for each individual sequela are available in Additional file 2.

The eight studies were conducted in Europe between 2004 and 2019; four in Sweden, three in the UK and one in the Netherlands. The sequelae investigated were mostly gastrointestinal, with some non-gastrointestinal symptoms such as joint pain and eye pain and most recruited cases were adults. This was in contrast to studies in non-industrialised countries which focused on growth, nutrition and cognitive detriment in children.

The most frequently investigated sequelae are listed in Table 2 and included diarrhoea, abdominal pain, vomiting, fatigue, joint pain, eye pain and headache.

The most common reported long-term sequelae were diarrhoea (25%), abdominal pain (25%), nausea (24%), fatigue (24%) and headache (21%). The distribution of gastrointestinal manifestations and non-gastrointestinal manifestations reported is shown in Fig. 2.

#### Subgroup analysis

Table 3 shows the pooled estimates for the prevalence of post-*Cryptosporidium* sequelae by time period post-infection. With the exception of eye pain and headache, all sequelae were more frequently reported within 6 months of *Cryptosporidium* infection.

In all eight studies included in the quantitative analysis, species identification of *Cryptosporidium* had been performed. Four were outbreak cohort follow-up studies so contained only one species (three contained *C. hominis* cases exclusively and one contained *C. parvum* exclusively). The other four studies contained both species; one of these four also contained a small number of other species (17/271 cases), but because of the low numbers, these have not been considered here. Figure 3 shows the pooled estimates for the prevalence of post-*Cryptosporidium* sequelae by *Cryptosporidium* species. Overall, long-term sequelae were more prevalent following infection with *C. hominis*, with only weight loss and blood in stool being more prevalent following infection with *C. parvum*. IBS was reported in 11% of cases, however, it should be noted that data for this outcome were only available from 2 studies, one of which only studied *C. parvum* cases.

#### Sequelae risk

Five of the 8 qualitative synthesis studies included a control group. A limited evaluation of risk of individual sequelae using the five case-control studies available was undertaken [4, 15, 17, 19, 20]. Data were available for 10 sequelae (Table 4).

**Fig. 4:**
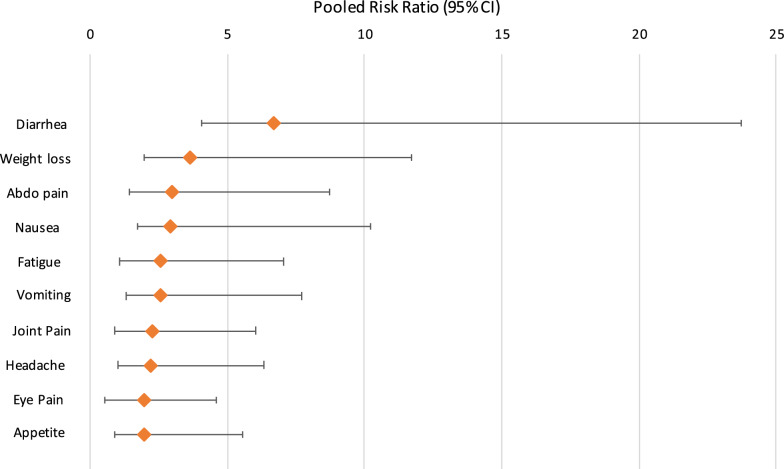
Pooled risk ratio of individual post-*Cryptosporidium* sequelae showing 95% confidence intervals

Individuals were 6 times more likely to report chronic diarrhoea and weight loss up to 28 months after a *Cryptosporidium* infection than controls. Long-term abdominal pain, loss of appetite, fatigue, vomiting, joint pain, headache and eye pain were also 2–3 times more likely following a *Cryptosporidium* infection (Fig. 4).

To view the PRISMA checklist relating to this work, please see Additional file 3.

## Discussion

Of the 15 studies investigating long-term sequelae, just over half were set in industrialised countries. In contrast to those in non-industrialised settings, these involved mainly adult cases, with the inclusion of some children. Half were outbreak cohort studies, with the rest involving sporadic community cases. Studies from non-industrialised countries involved exclusively children, reflecting the greater clinical importance and recognition of paediatric infection in such settings. In industrialised countries there is more focus on detecting sporadic community cases of cryptosporidiosis in all age groups, partly in order to facilitate early detection of community outbreaks, for example from drinking water, swimming pools, or other environmental sources. The studies in non-industrialised countries also differed in that the children were recruited and tested as part of the specific studies, whilst the studies in industrialised countries relied on cases initially diagnosed routinely.

The eight studies suitable for inclusion in the quantitative analysis were all carried out in just three countries in Europe (UK, Sweden and the Netherlands), where species data are routinely generated and all except one [11] were relatively recent, dated between 2013–2019. In many non-industrialised countries, or in earlier European studies, species identification would not be routinely performed, and this is reflected in the study data. The geographical reach of the eight studies is somewhat limited, since they were all located in northwest Europe. Only five were case-control studies, and of these, only two included both *C. hominis* and *C. parvum*, with the other three limited to studying *C. hominis* alone following outbreaks. Since the bulk of the cryptosporidiosis burden is found in low-income countries, there is a need in future to conduct similar quantitative evaluations using data from developing countries, where obtaining suitable data may be more challenging.

There were some limitations to this review. The role of genotype in long-term outcomes could not be explored. Typing was undertaken by *gp60* sequencing in three of the studies but was either not analysed with symptoms data [16], or was an outbreak where all had the same subtype [4, 18]. There were insufficient data to compare between studies. Another limitation was that since not all cases were necessarily tested for all gastrointestinal pathogens, or the results of such tests were not stated, long-term sequelae identified cannot be proven to be *Cryptosporidium*-specific and not due to other infectious agents.

Most of the studies examined quantitatively were concentrated on adult individuals, whereas cryptosporidiosis is commonest in young children. This over-representation of adults results from the fact that several of the studies followed large waterborne outbreaks involving many adults, rather than sporadic cases. Identifying and defining sometimes rather non-specific sequelae is more difficult in very young children. However, a study by Carter et al. [21] of sporadic cases did include children, and in fact this study found that the proportion developing IBS or IBS-like symptoms was higher in children than in adults, with 78% reporting it among 5–17 years-old and 63% at 6 months to 4 years-old.

The results indicate that sequelae are frequently reported after cryptosporidiosis lasting up to at least 2 years. Only one study investigated cases for longer, up to 36 months [11]. For both main infecting species, sequelae occur, but there are differences in the frequency of each depending on the species. Following the publication of the first study in 2004 [15], the evidence base surrounding post-*Cryptosporidium* infection sequelae has continued to expand [16–21]. Gastrointestinal sequelae such as continuing diarrhoea, nausea and abdominal pain appear particularly common, each reported by around a quarter of cases up to 36 months post-infection, with analysis of the case-control studies finding that persistent diarrhoea is around six times more likely than in controls and weight loss over three times more likely over 28 months. Fatigue and headache were also commonly reported and occurred in the case-control studies between two-three times more commonly in cases than controls over the same time period. Overall, the most commonly reported long-term sequelae were diarrhoea (25%), abdominal pain (25%), nausea (24%), fatigue (24%) and headache (21%). Where it was investigated, there was evidence that symptoms meeting the definition for IBS were described just over 10% of cases up to 36 months.

## Conclusions

This is the first systematic review of the long-term sequelae of cryptosporidiosis. The proportion of cases self-reporting sequelae post-infection has been estimated and estimates of risk of specific sequelae presented. Risk factors for sequelae were less well identified. A better understanding of the long-term outcomes of cryptosporidiosis is valuable to inform the expectations of clinicians and their patients and public health policy makers regarding the control and prevention of this infection.

## Supplementary information

**Additional file 1: Table S1.** Full electronic search strategies. **Table S2.** Newcastle-Ottawa quality assessment scale.

**Additional file 2.** Data for individual sequelae.

**Additional file 3.** PRISMA checklist.

## Data Availability

All data generated or analysed during this study are included in this published article and its additional files.
