# Supplementary material for: Health sequelae of human cryptosporidiosis in industrialised countries: a systematic review
Source: Parasit Vectors. 2020 Sep 4;13:443. doi: 10.1186/s13071-020-04308-7 (PMC7650228; doi:10.1186/s13071-020-04308-7)
Supplement: Supplementary file 1 — Additional file 1: Table S1. Full electronic search strategies. Table S2. Newcastle-Ottawa quality assessment scale. [file 13071_2020_4308_MOESM1_ESM.docx]

**Additional file 1: Table S1. Full electronic search strategies**

| **Search term** | **Synonym/Alternative** | **MeSH term** | **Truncation** |
| --- | --- | --- | --- |
| *Cryptosporidium* | *Cryptosporidium parvum*,  *Cryptosporidium hominis, C. parvum, C. hominis* | *Cryptosporidium* | Cryptosporid* |
| Cryptosporidiosis | *Cryptosporidium* infection | Cryptosporidiosis |  |
| Health sequelae | Concomitant conditions,  Concomitant disease, Sequels, Sequela, Associated conditions,  Associated disease,  Health consequences, Post-infection symptoms, Post-infectious, Chronic, Long-term, Morbidity | - | Sequel*  Post-infecti* |

PubMed = 1,251

| **Date of Search** | **#** | **Search query** | **Limits** | **Hits** |
| --- | --- | --- | --- | --- |
| 26/07/2019 | 1 | Cryptosporid*[Title/Abstract] AND Sequel*[Title/Abstract] |  | 20 |
| 26/07/2019 | 2 | Cryptosporid*[Title/Abstract] AND Post-infecti*[Title/Abstract] |  | 114 |
| 26/07/2019 | 3 | Cryptosporid*[Title/Abstract] AND ("long-term"[Title/Abstract] OR chronic[Title/Abstract]) |  | 696 |
| 26/07/2019 | 4 | Cryptosporid*[Title/Abstract] AND Complications[Title/Abstract] |  | 92 |
| 26/07/2019 | 5 | Cryptosporid*[Title/Abstract] AND ("Associated conditions"[Title/Abstract] OR "Associated disease"[Title/Abstract] OR "Concomitant conditions"[Title/Abstract] OR "Concomitant disease"[Title/Abstract] OR "Health consequences"[Title/Abstract]) |  | 17 |
| 26/07/2019 | 6 | Cryptosporid*[Title/Abstract] AND Morbidity[Title/Abstract] |  | 312 |

ProQuest = 2,161

| **Date of Search** | **#** | **Search query** | **Limits** | **Hits** |
| --- | --- | --- | --- | --- |
| 26/07/2019 | 1 | noft(Cryptosporid*) AND noft(Sequel*) | Humans  Scholarly Journals | 11 |
| 26/07/2019 | 2 | noft(Cryptosporid*) AND noft(Post-infecti*) | Humans  Scholarly Journals | 28 |
| 26/07/2019 | 3 | noft(Cryptosporid*) AND (noft("long-term") OR noft(chronic)) | Humans  Scholarly Journals | 560 |
| 26/07/2019 | 4 | noft(Cryptosporid*) AND noft(Complications) | Humans  Scholarly Journals | 1250 |
| 26/07/2019 | 5 | noft(Cryptosporid*) AND (noft("Associated conditions") OR noft("Associated disease") OR noft("Concomitant conditions")OR noft("Concomitant disease") OR noft("Health consequences")) | Humans  Scholarly Journals | 16 |
| 26/07/2019 | 6 | noft(Cryptosporid*) AND noft(Morbidity) | Humans  Scholarly Journals | 296 |

Web of Science = 3,227

| **Date of Search** | **#** | **Search query** | **Limits** | **Hits** |
| --- | --- | --- | --- | --- |
| 26/07/2019 | 1 | **TOPIC:** (Cryptosporid* AND Sequel*)  *Databases= WOS, BCI, KJD, MEDLINE, RSCI, SCIELO Timespan=All years* |  | 29 |
| 26/07/2019 | 2 | **TOPIC:** (Cryptosporid* AND Post-infecti*)  *Databases= WOS, BCI, KJD, MEDLINE, RSCI, SCIELO Timespan=All years* |  | 127 |
| 26/07/2019 | 3 | **TOPIC:** (Cryptosporid* AND ("long-term" OR chronic))  *Databases= WOS, BCI, KJD, MEDLINE, RSCI, SCIELO Timespan=All years* |  | 1069 |
| 26/07/2019 | 4 | **TOPIC:** (Cryptosporid* AND Complications)  *Databases= WOS, BCI, KJD, MEDLINE, RSCI, SCIELO Timespan=All years* |  | 1508 |
| 26/07/2019 | 5 | **TOPIC:** (Cryptosporid* AND ("Associated conditions" OR "Associated disease" OR "Concomitant conditions" OR "Concomitant disease" OR "Health consequences"))  *Databases= WOS, BCI, KJD, MEDLINE, RSCI, SCIELO Timespan=All years* |  | 25 |
| 26/07/2019 | 6 | **TOPIC:** (Cryptosporid* AND Morbidity)  *Databases= WOS, BCI, KJD, MEDLINE, RSCI, SCIELO Timespan=All years* |  | 469 |

**Table S2 - Newcastle-Ottawa quality assessment scale**

| **Studies** | **Selection** | **Comparability** | **Outcome** | **Total** |
| --- | --- | --- | --- | --- |
| **Cohort Studies** |  |  |  |  |
| Ajjampur *et al*, 2011 [31] | *** | ** | ** | 7 |
| Berkman *et al,* 2002 [32] | **** | ** | ** | 8 |
| Carter *et al*, 2019 [33] | *** | ** | ** | 7 |
| Guerrant *et al*, 1991 [35] | *** |  | *** | 6 |
| Insulander et al, 2013 [11] | *** |  | ** | 5 |
| Korpe *et al,* 2016 [36] | **** |  | ** | 6 |
| Phillips *et al*, 1992 [37] | *** | ** | *** | 8 |
| Stiff et al, 2017 [13] | *** | ** | ** | 7 |
|  | | | | |
| **Studies** | **Selection** | **Comparability** | **Exposure** | **Total** |
| **Case Control Studies** |  |  |  |  |
| Agnew et al, 1998 [30] | **** | ** | ** | 8 |
| Delahoy *et al*, 2018 [34] | **** | ** | * | 7 |
| Hunter et al, 2004 [10] | **** |  | ** | 6 |
| Igloi et al, 2018 [14] | **** | ** | * | 7 |
| Lilja *et al,* 2018 [15] | **** | ** | * | 7 |
| Rehn et al, 2015 [12] | **** | ** | * | 7 |
| Widerstrom et al, 2014 [4] | *** |  | ** | 5 |
