# Supplementary figures and images for: Health sequelae of human cryptosporidiosis in industrialised countries: a systematic review
Source: Parasit Vectors. 2020 Sep 4;13:443. doi: 10.1186/s13071-020-04308-7 (PMC7650228; doi:10.1186/s13071-020-04308-7)

**Additional file 2: Data for individual sequelae**


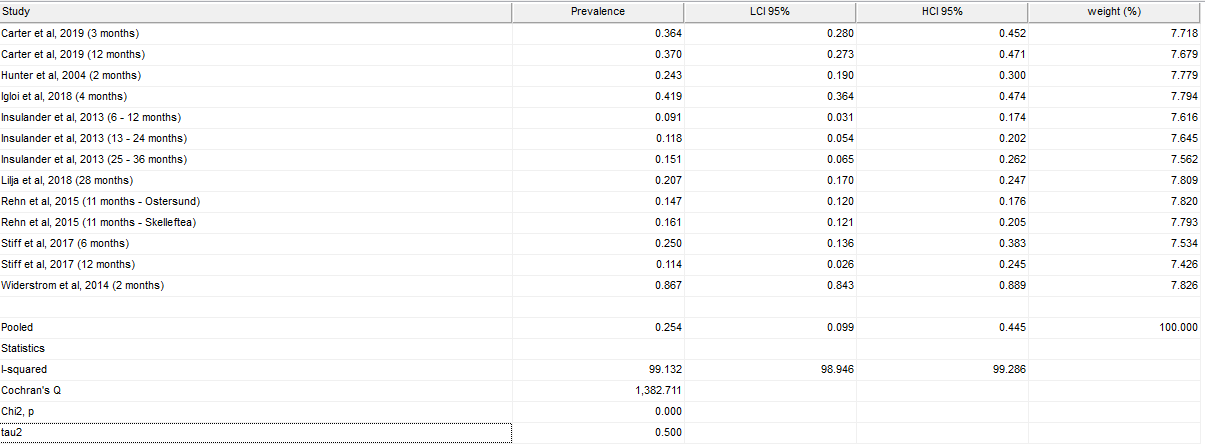

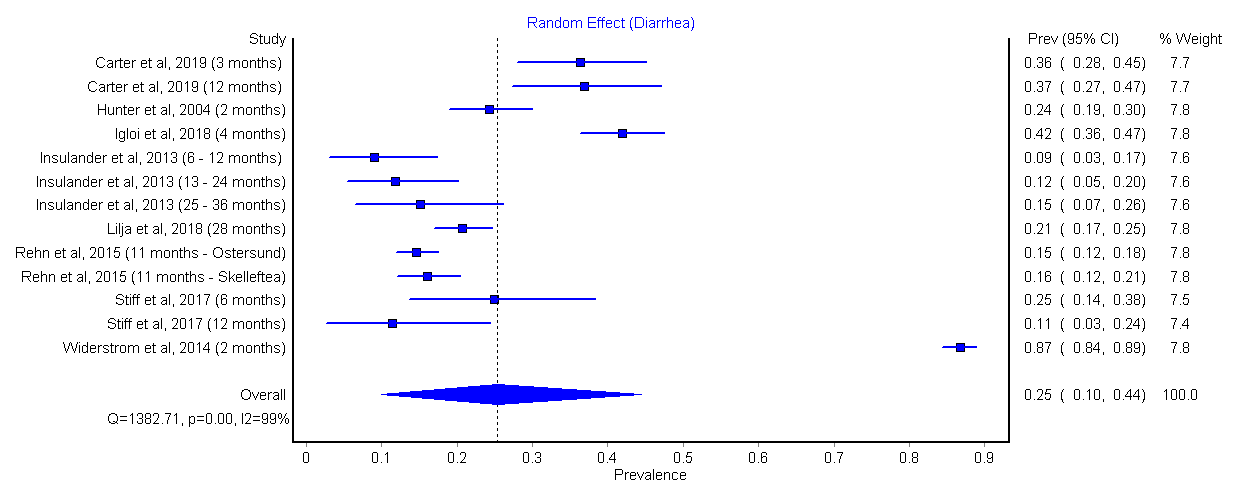


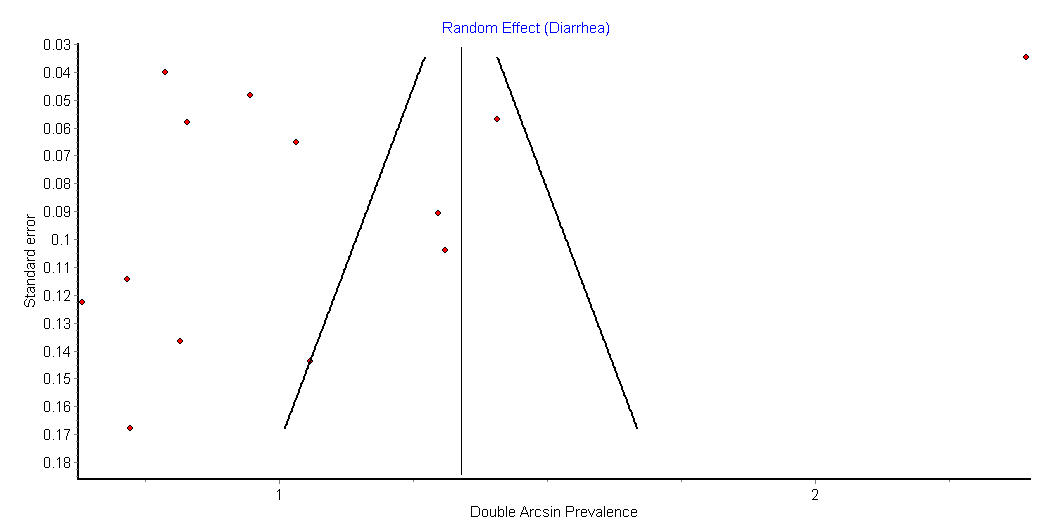


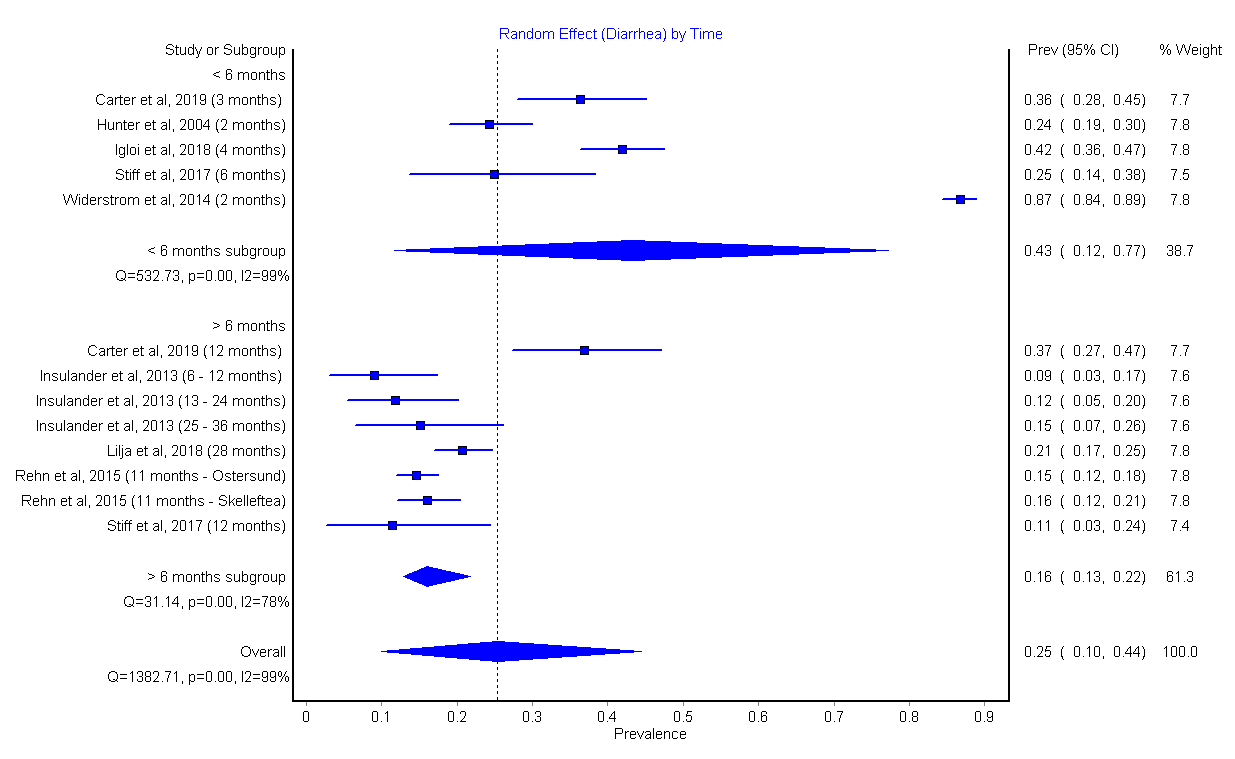


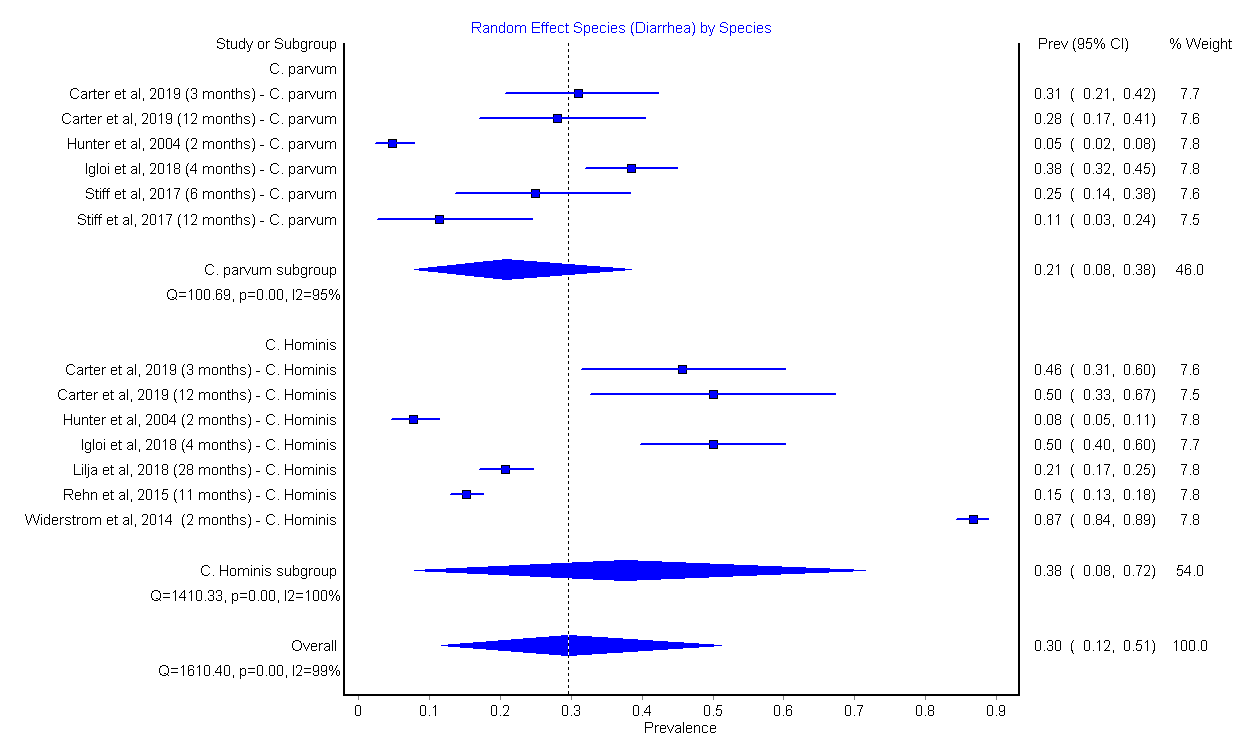


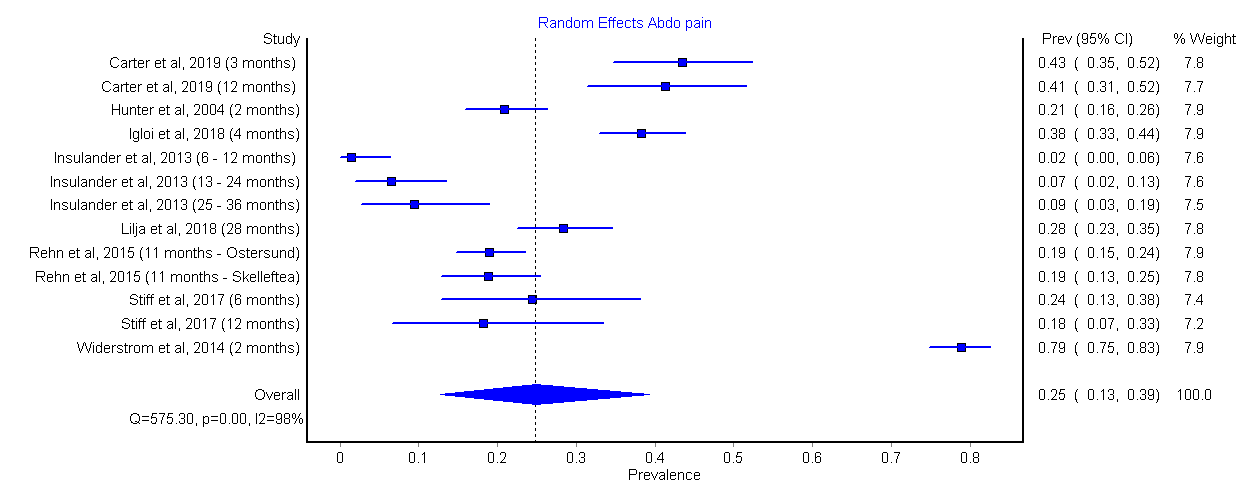


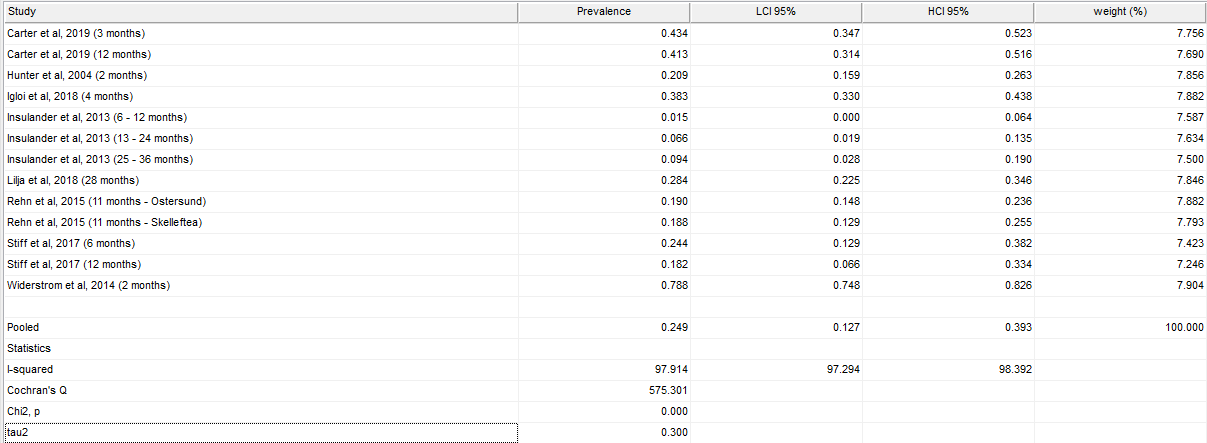


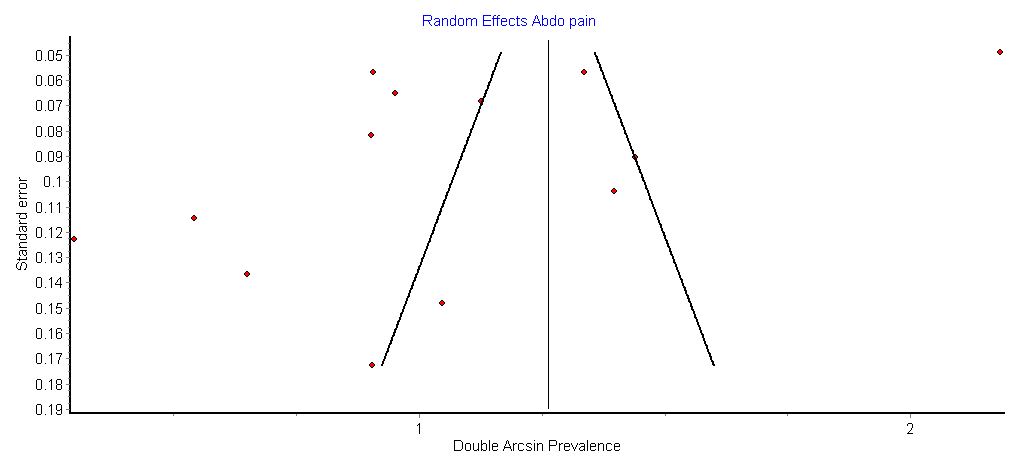


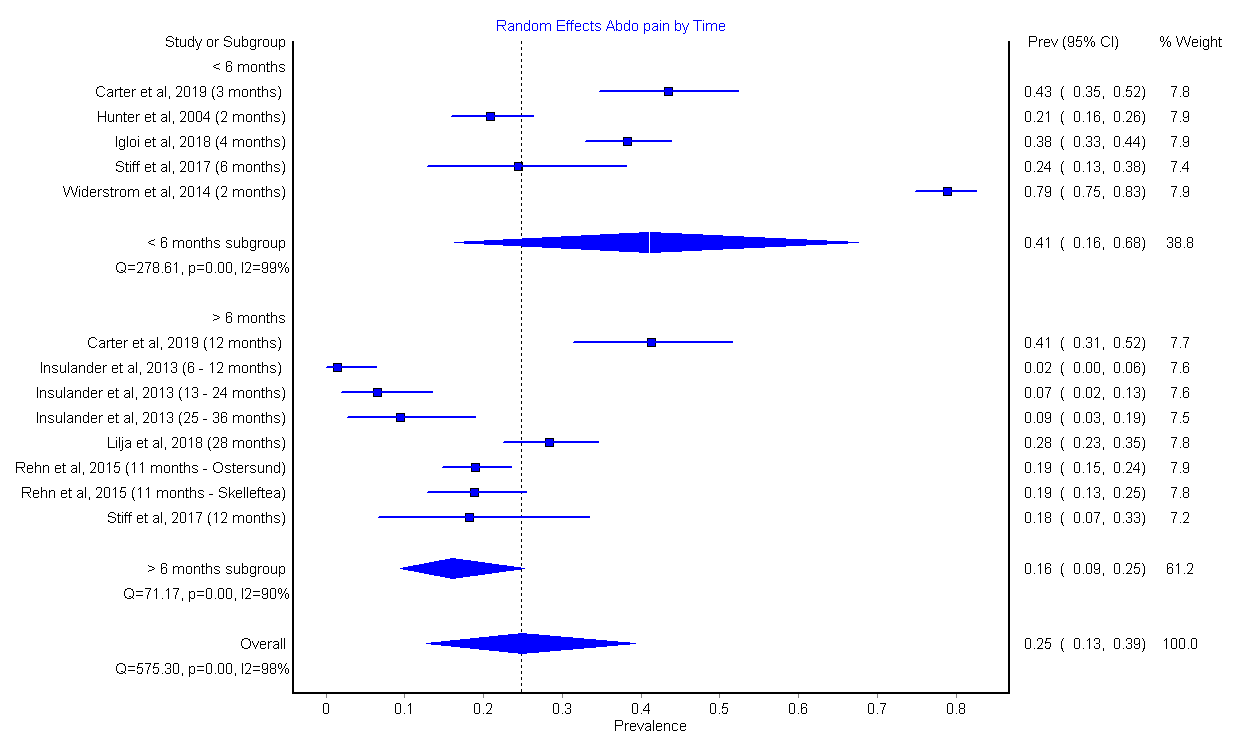


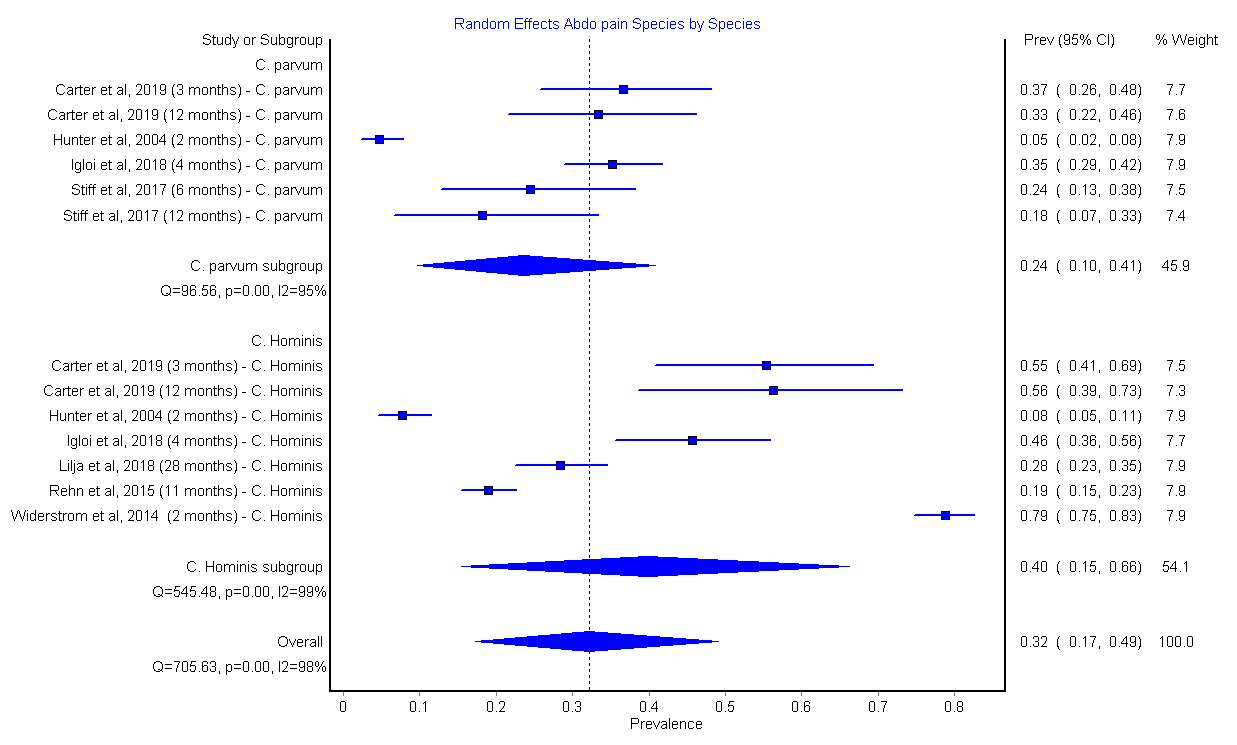


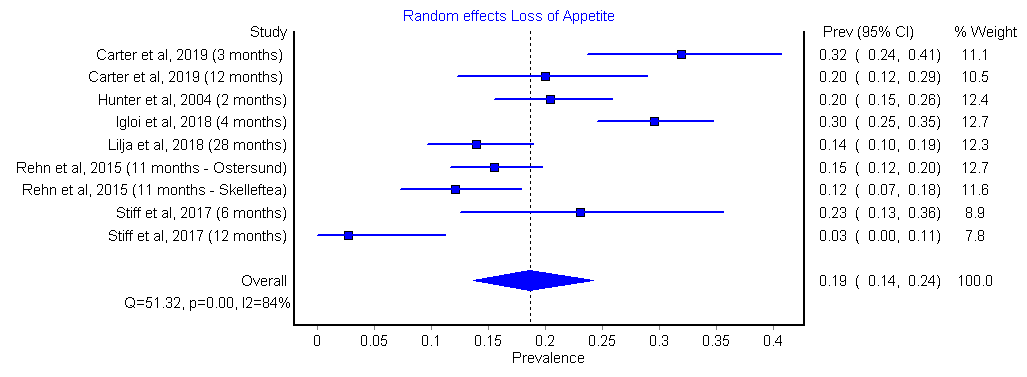


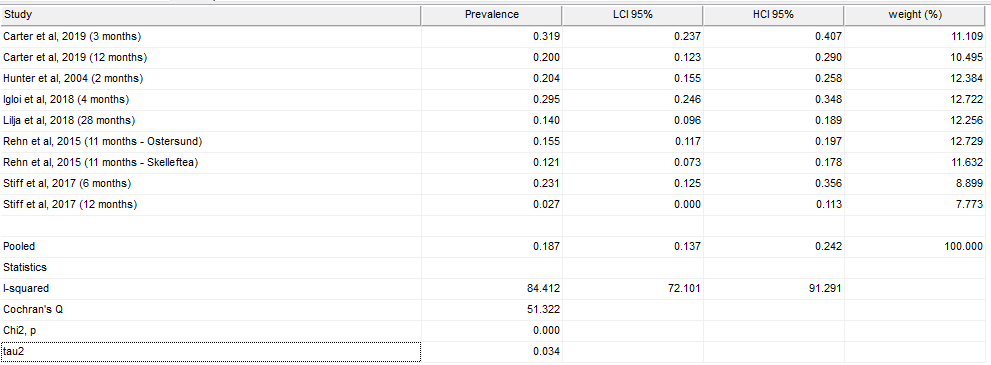


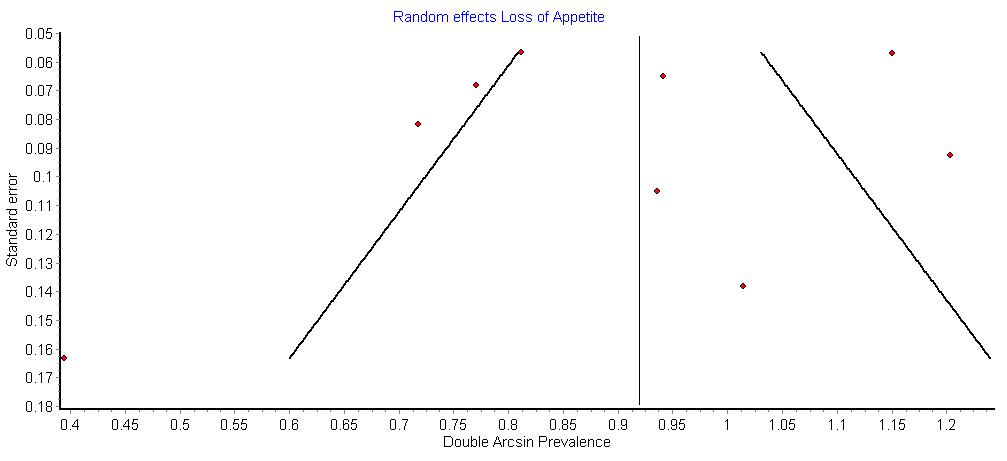


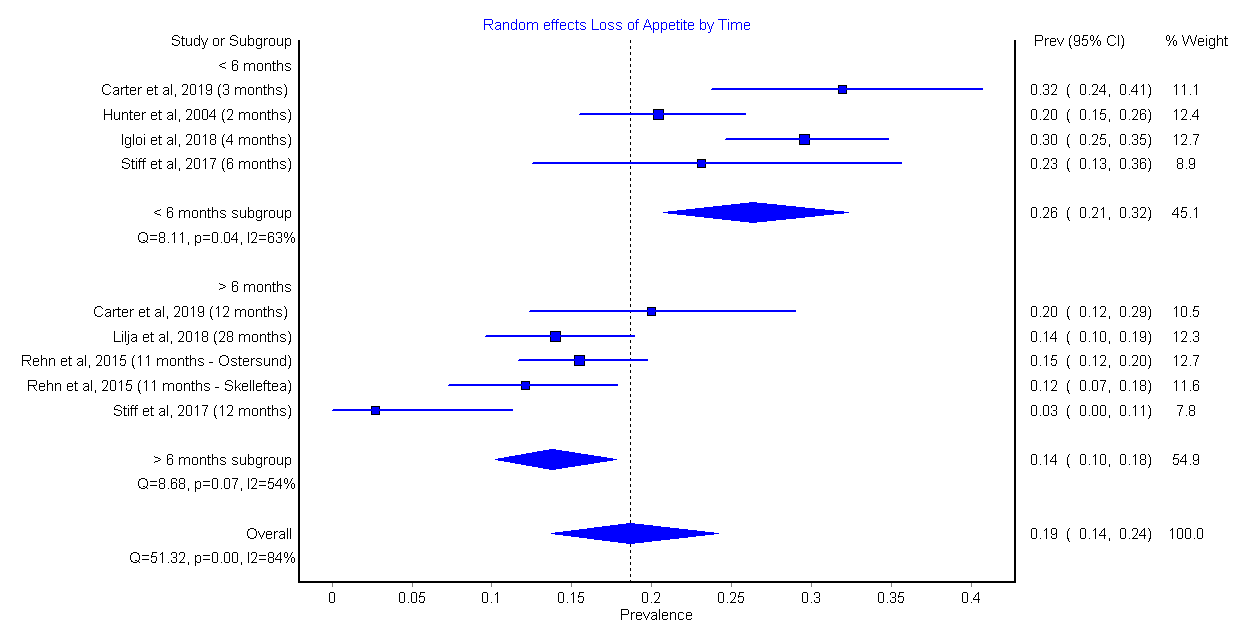


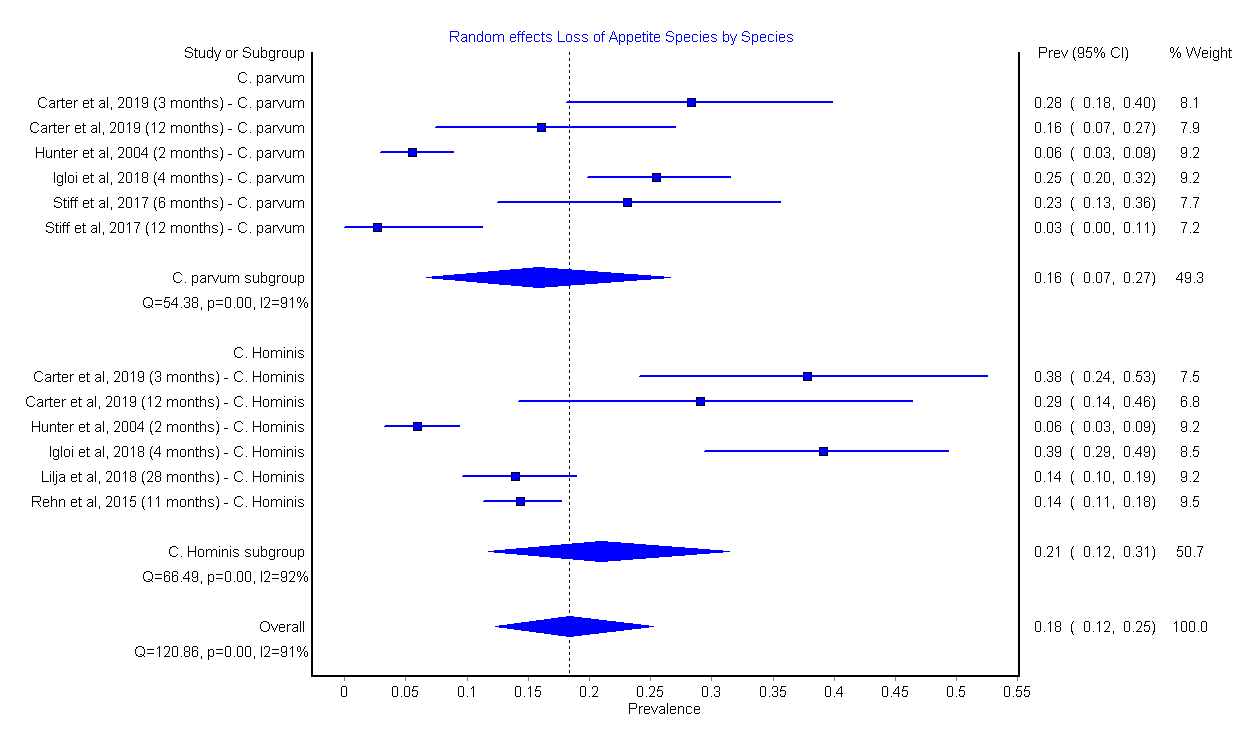


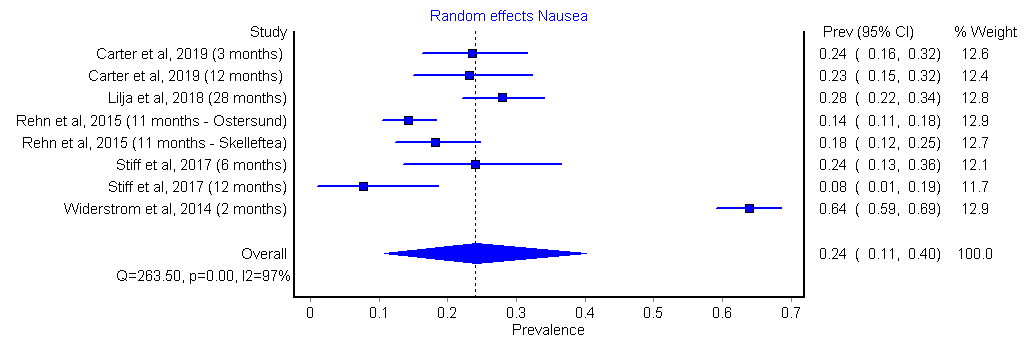


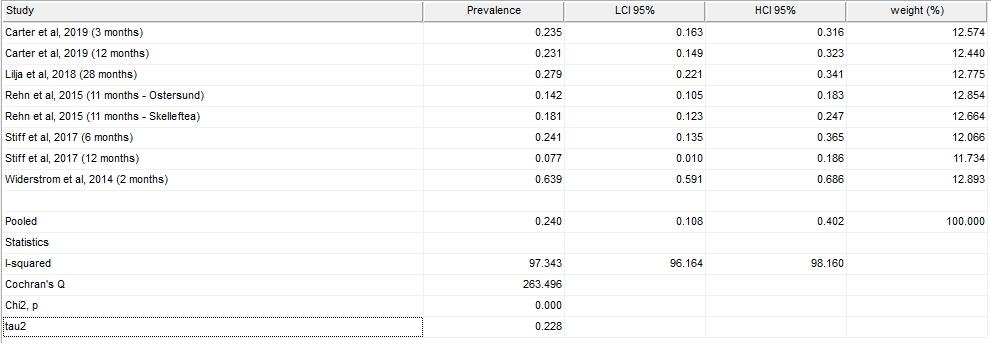


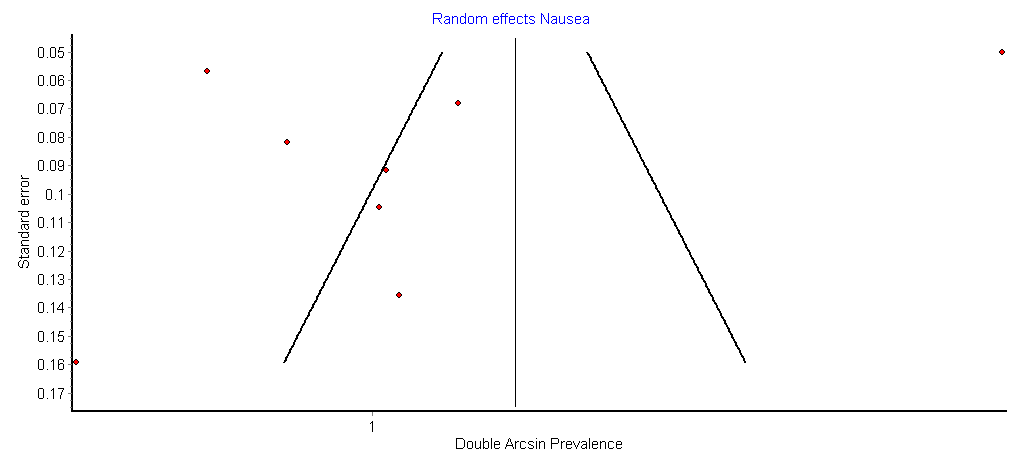


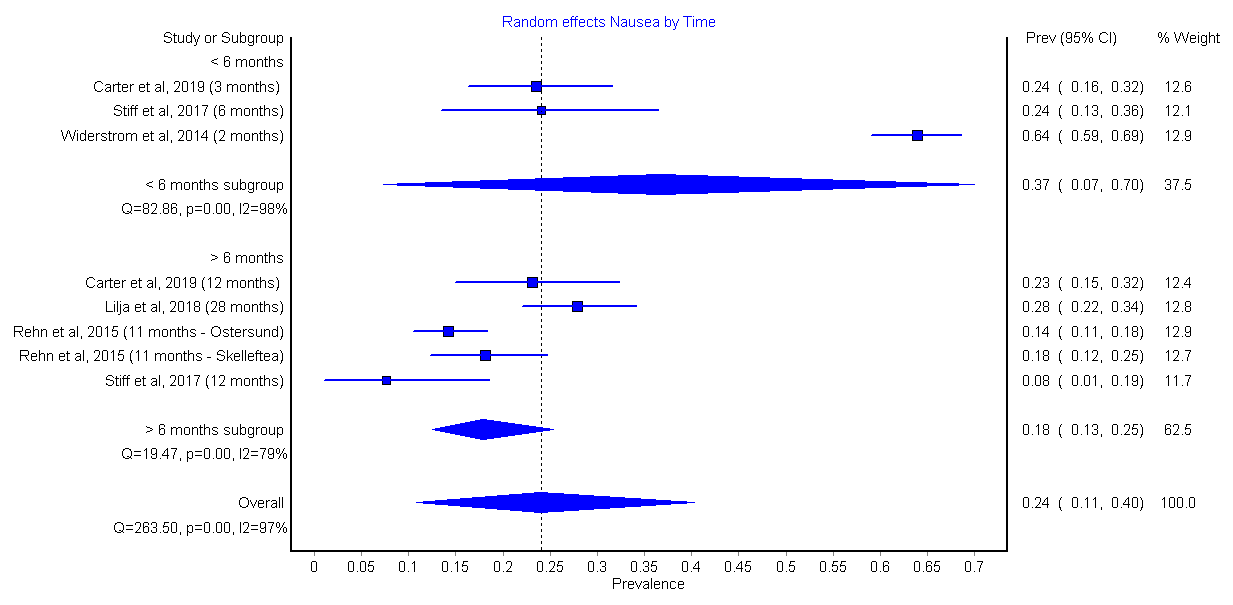


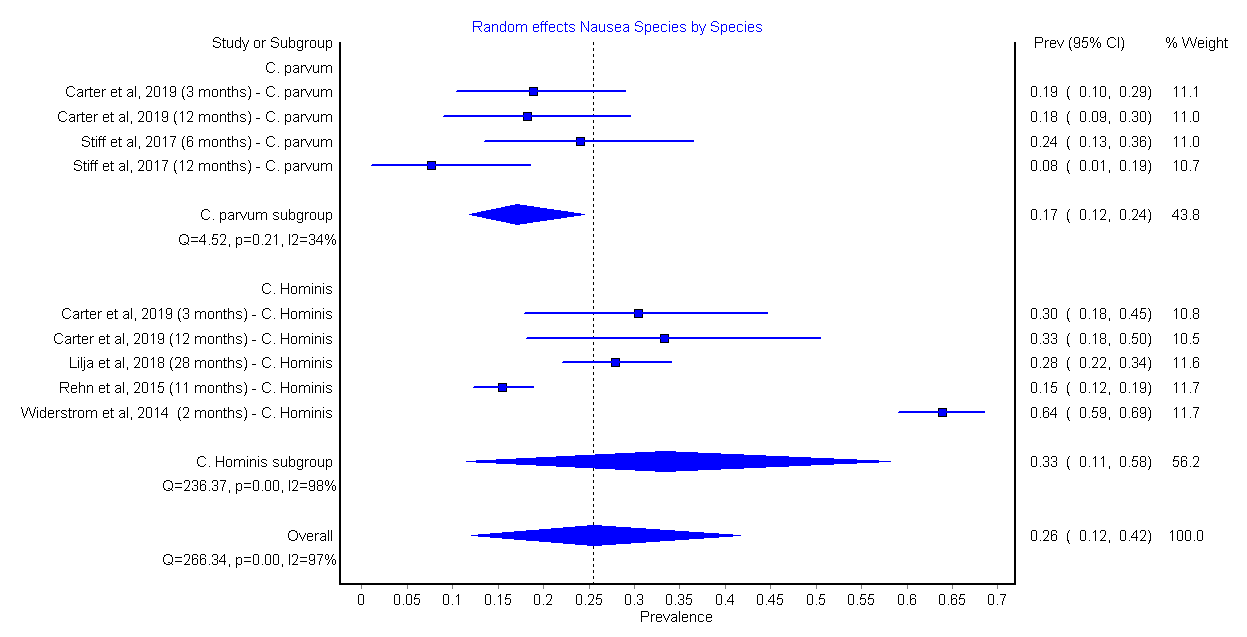


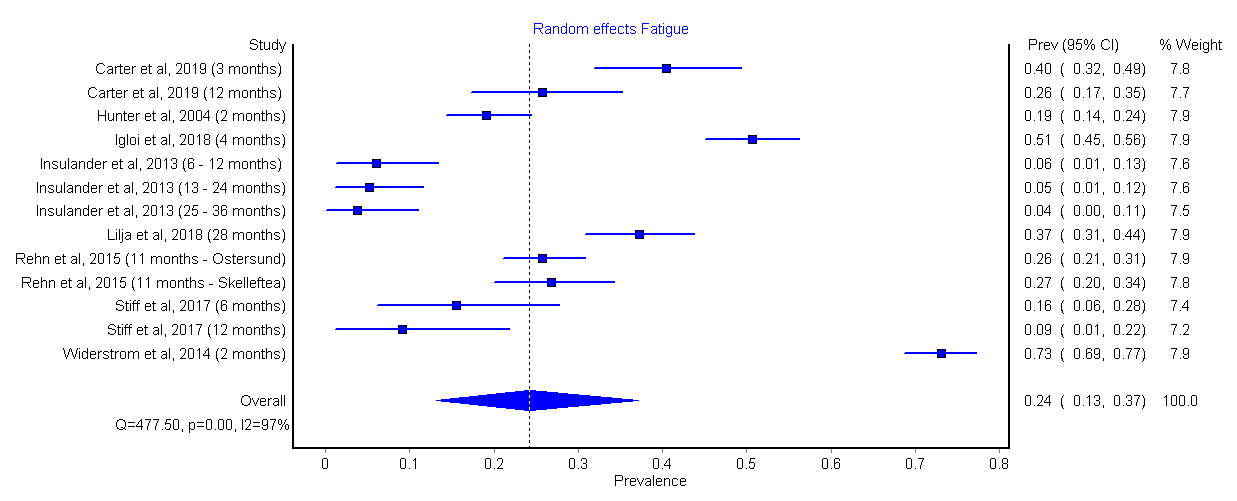


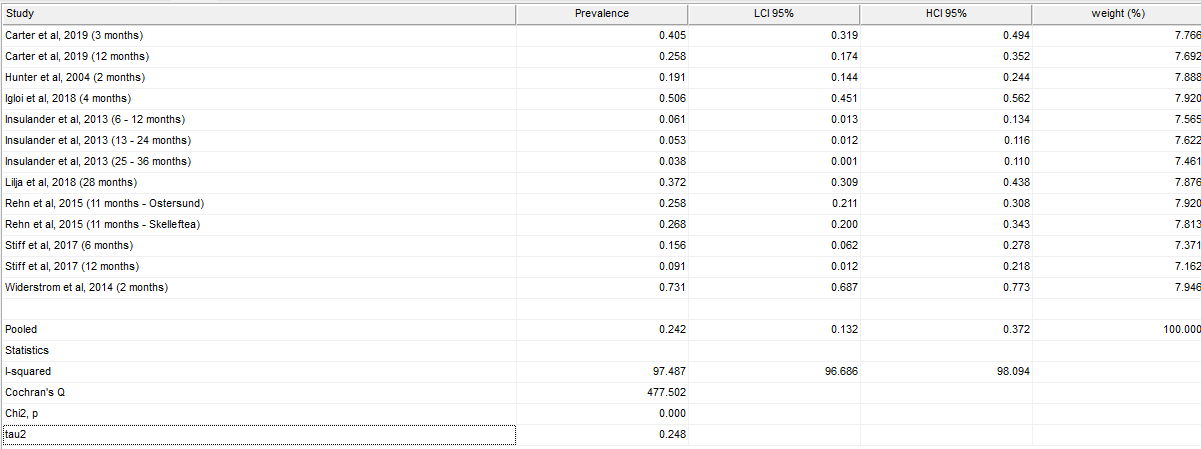


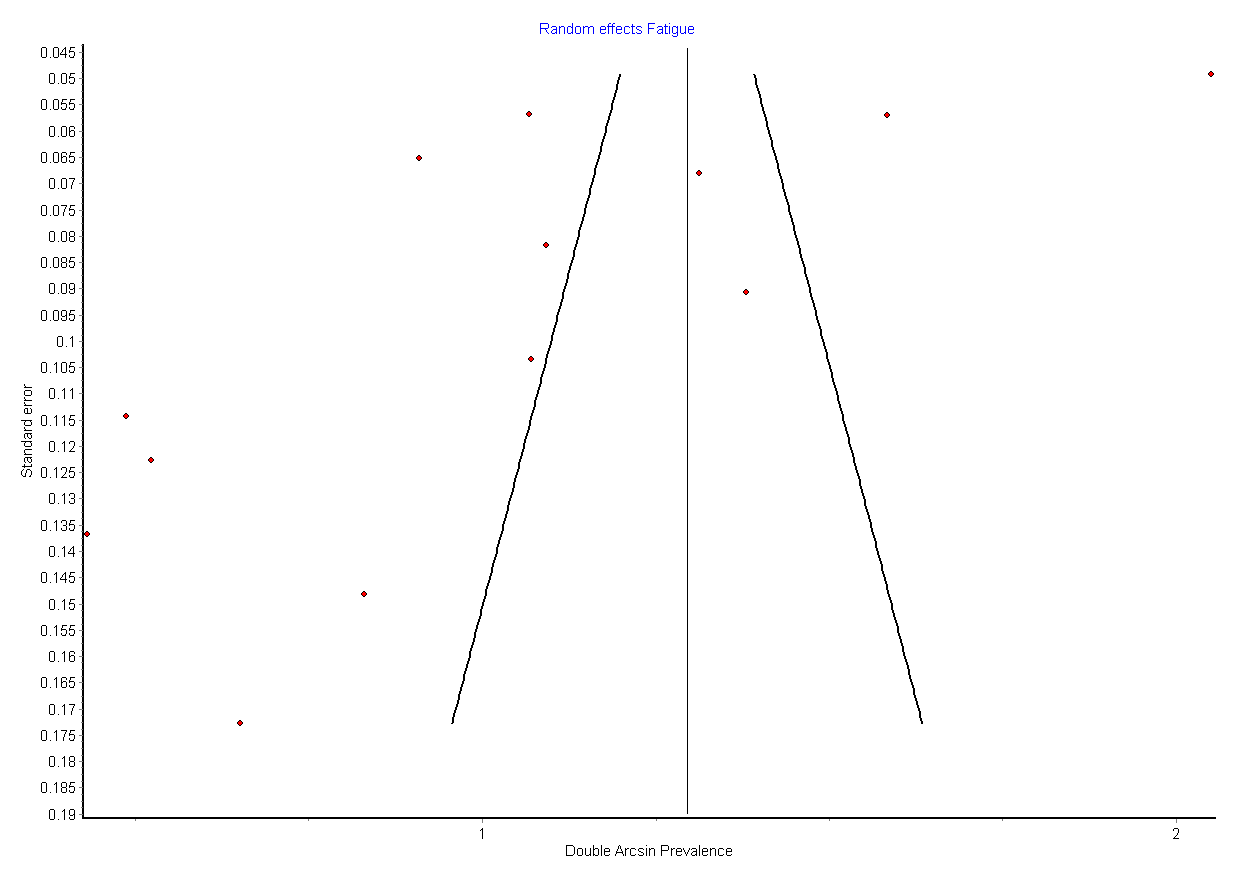


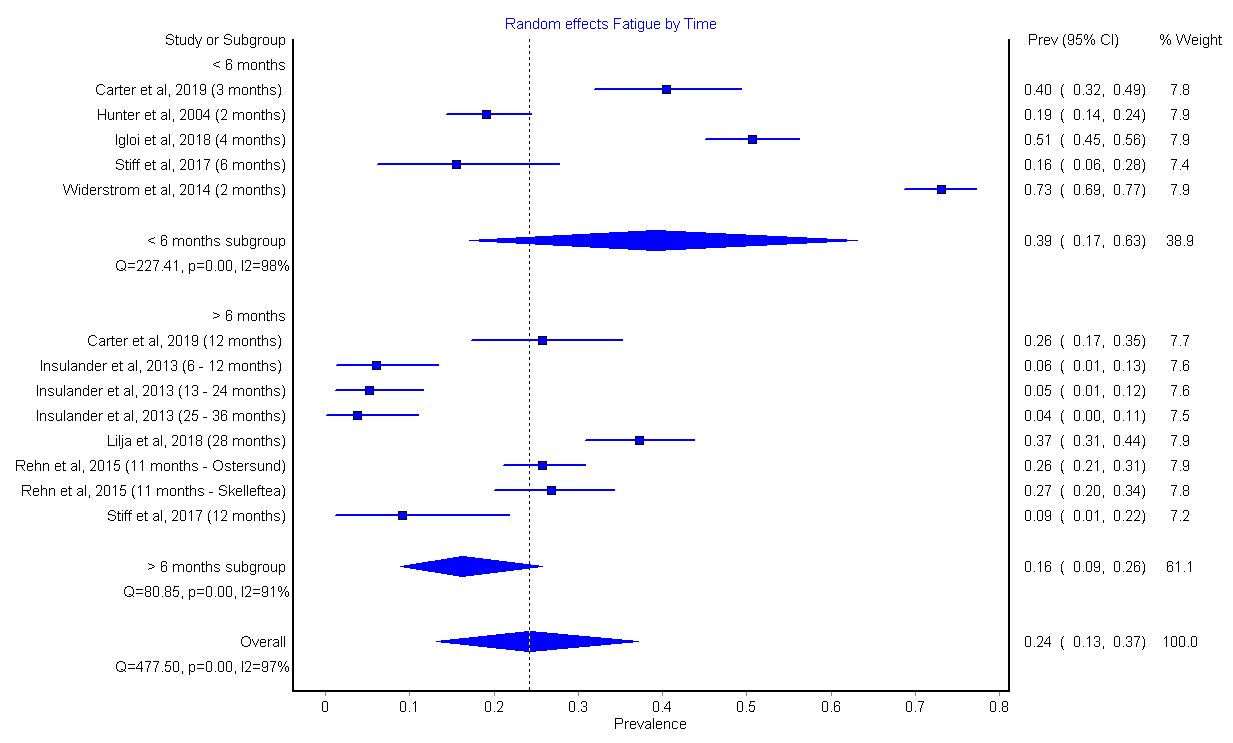


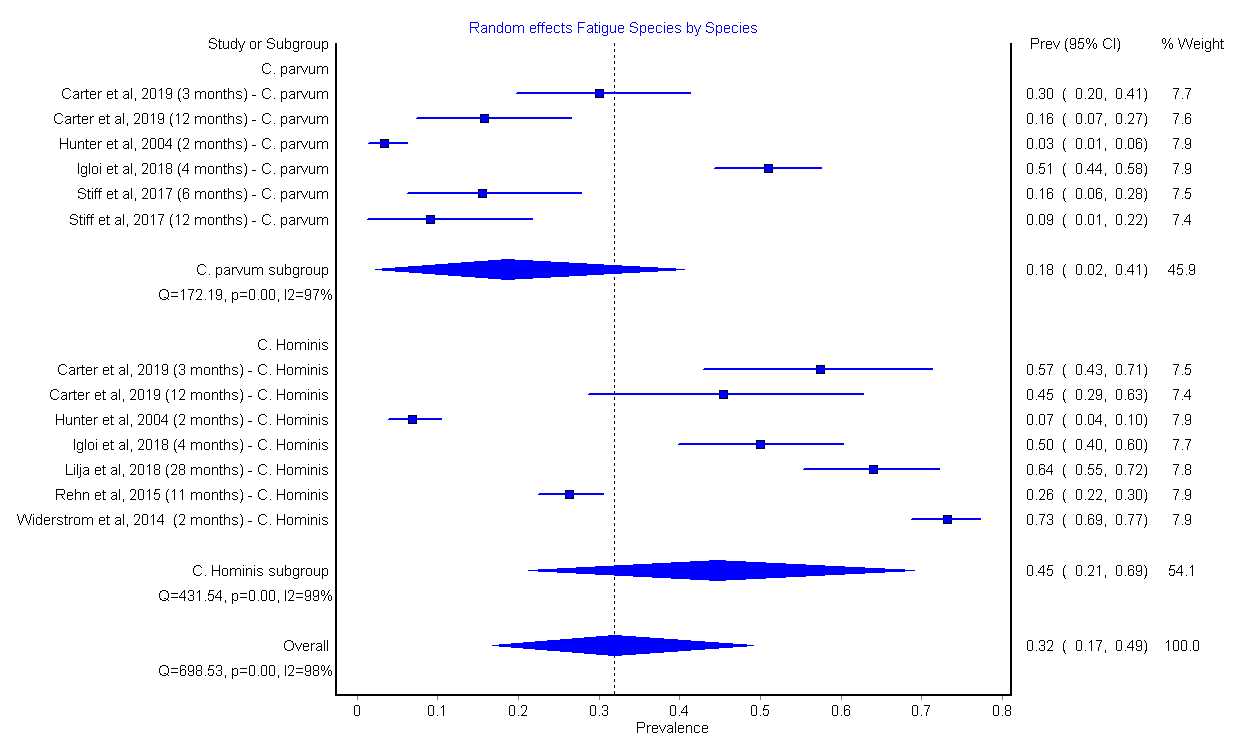


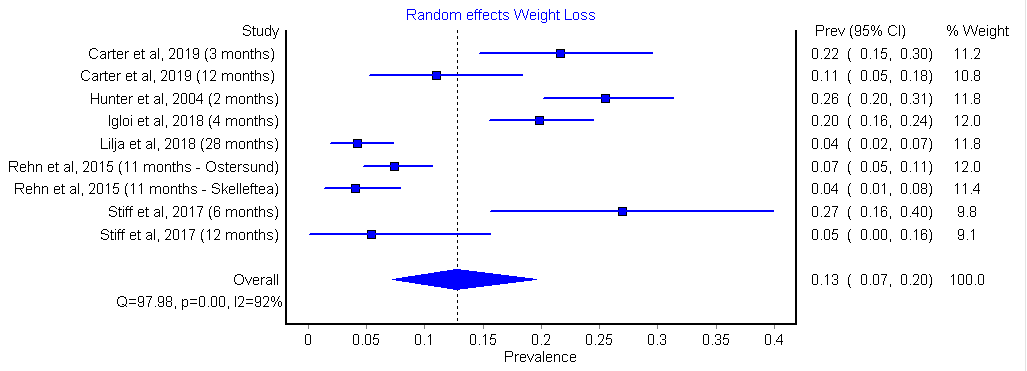


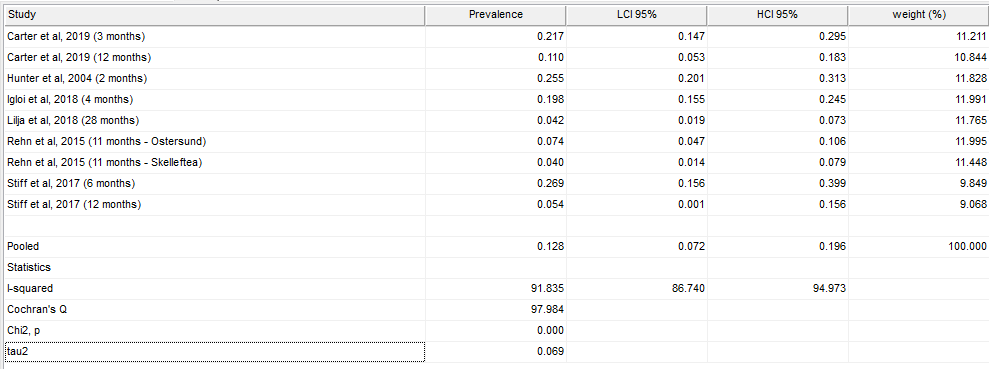


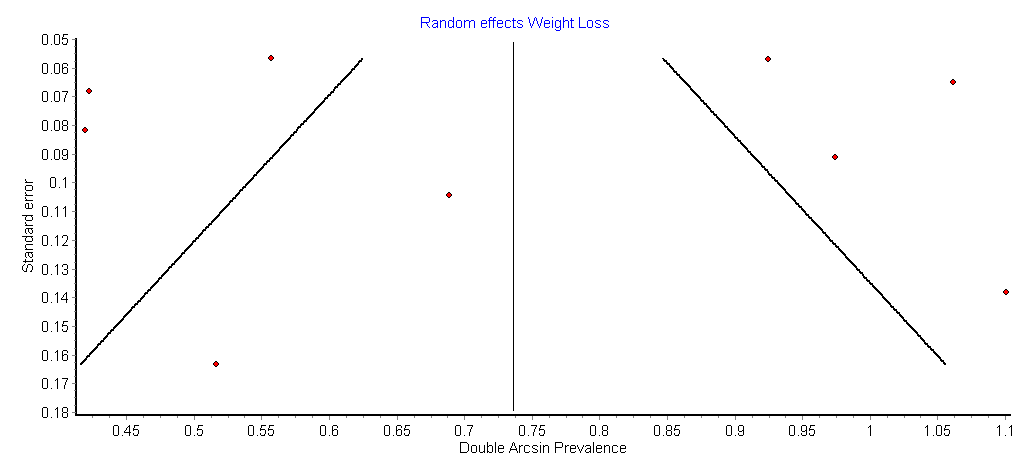


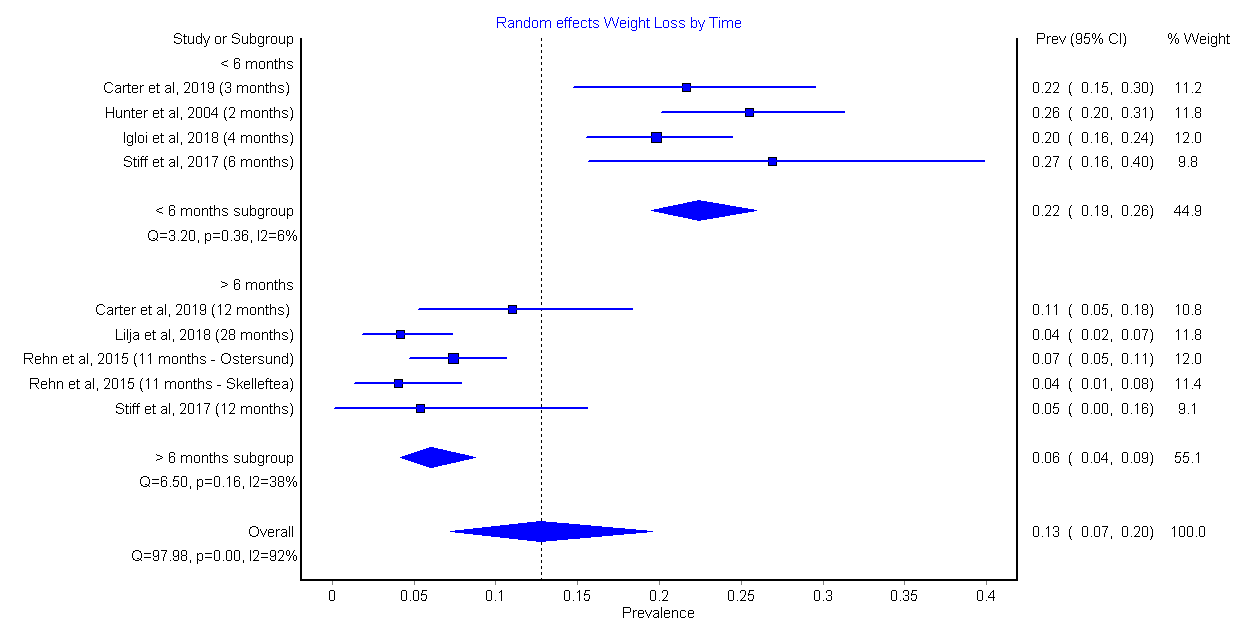


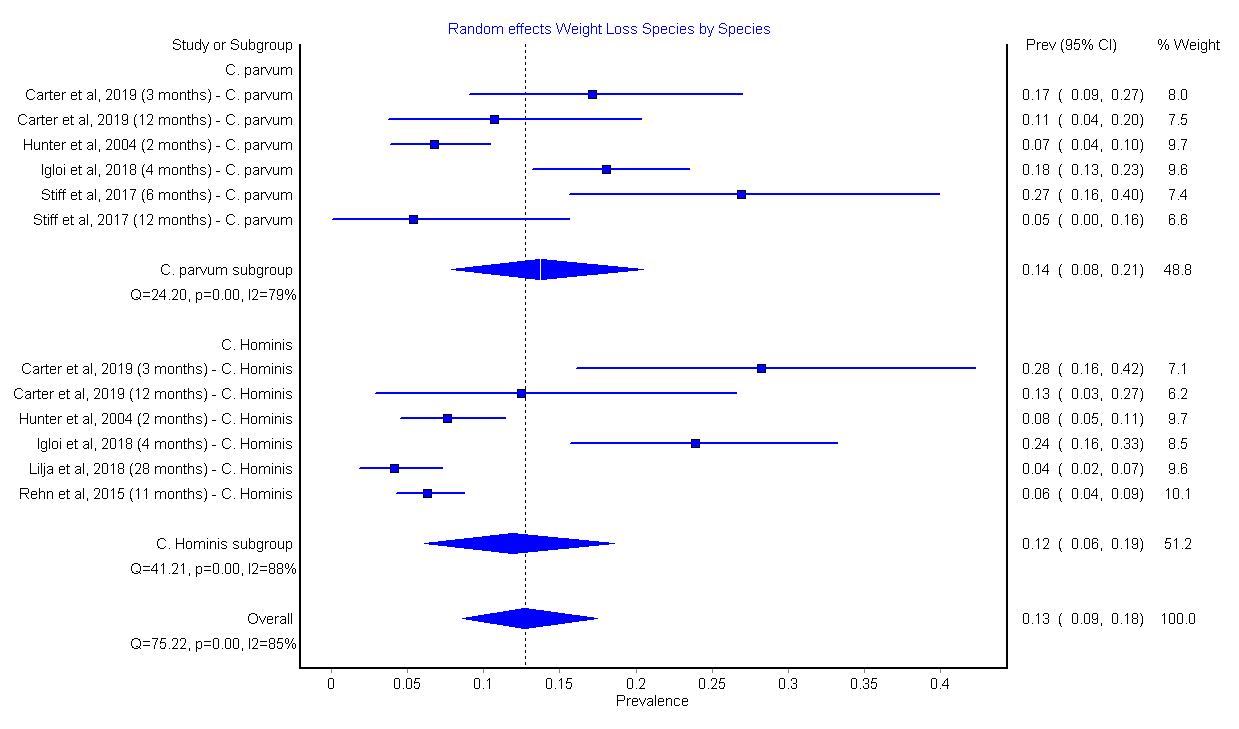


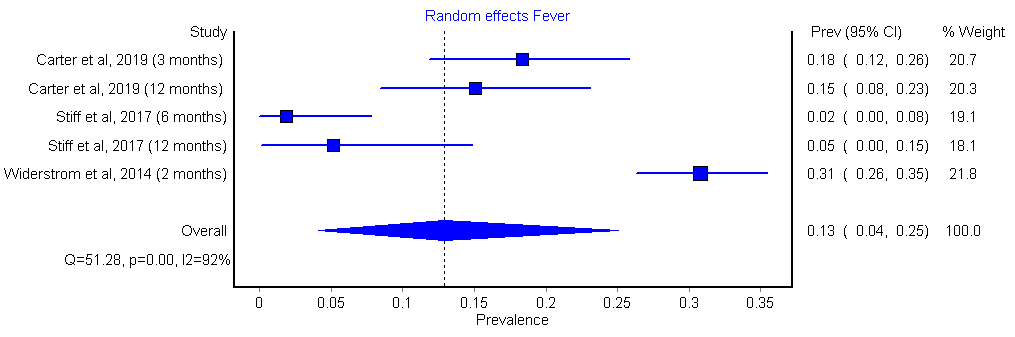


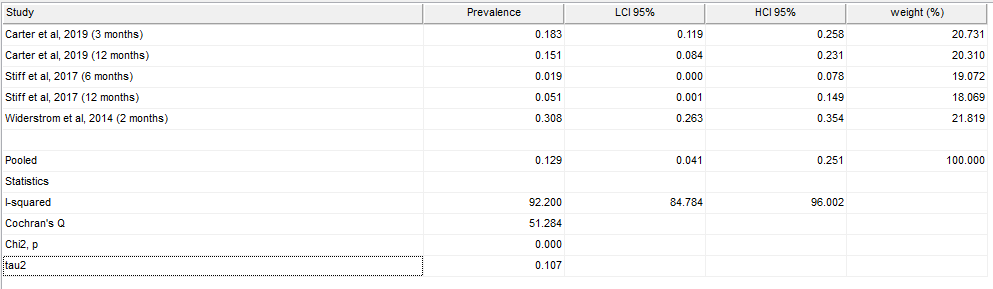


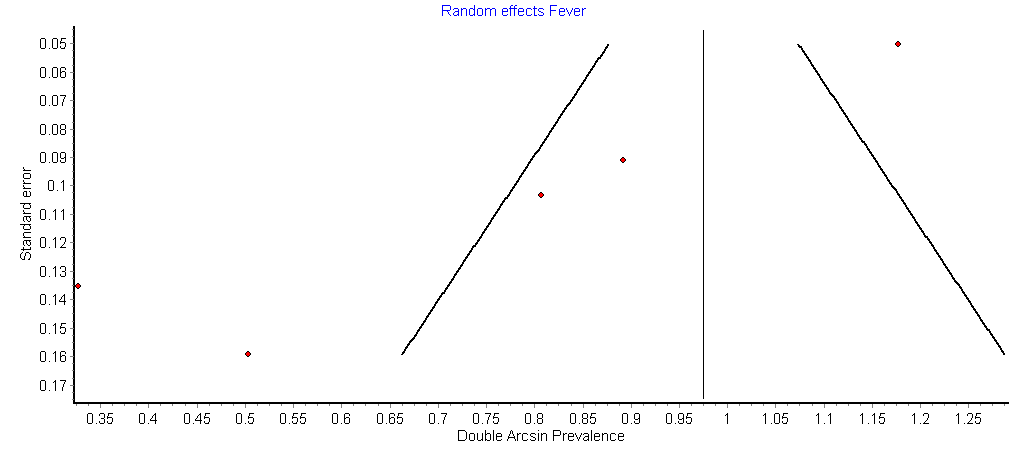


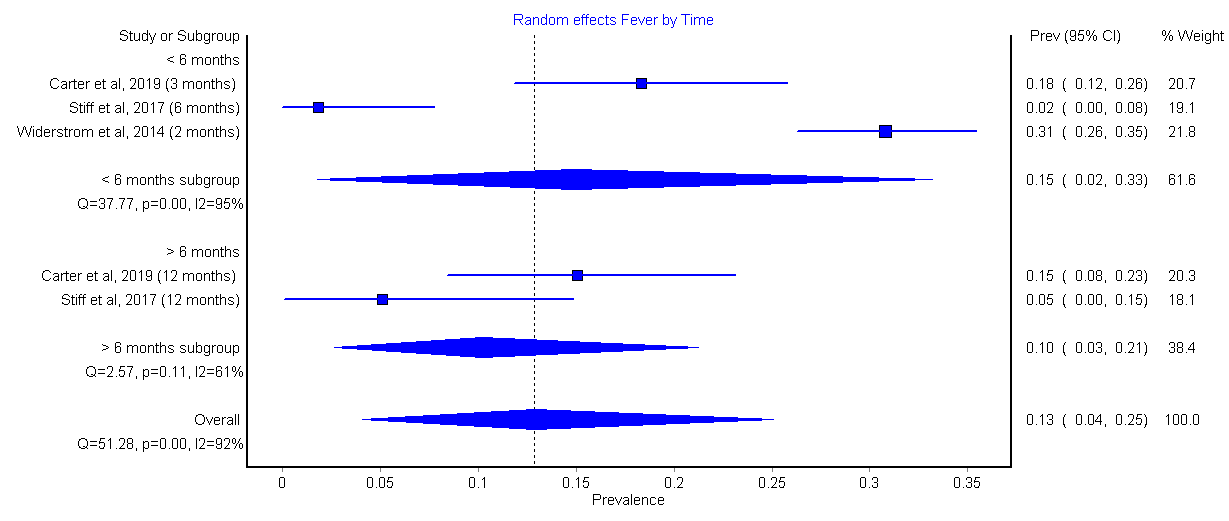


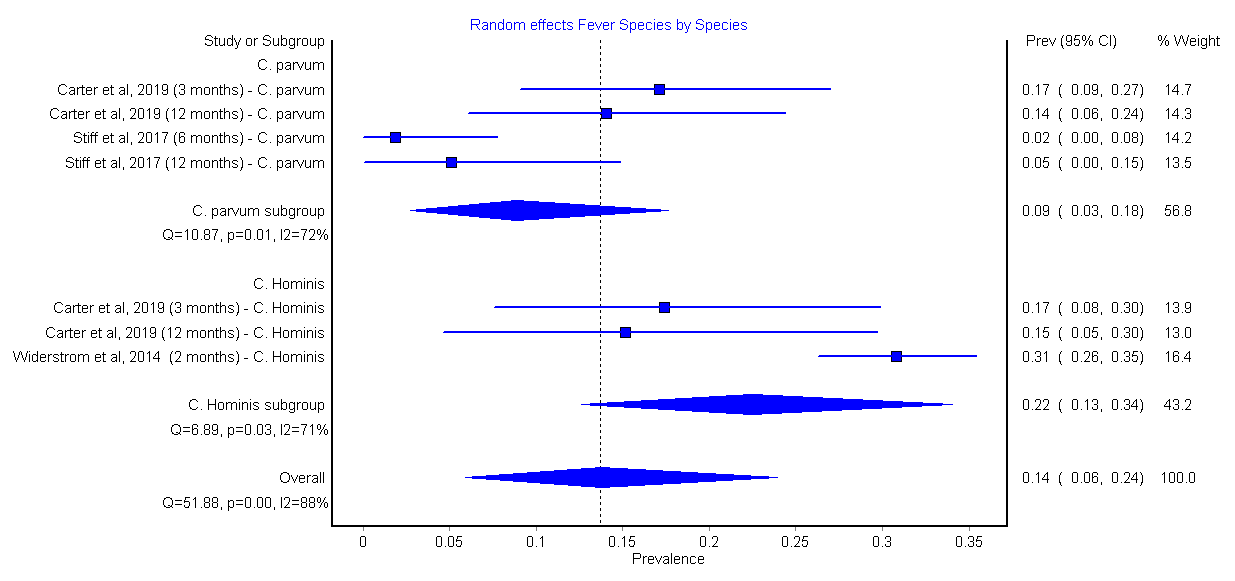


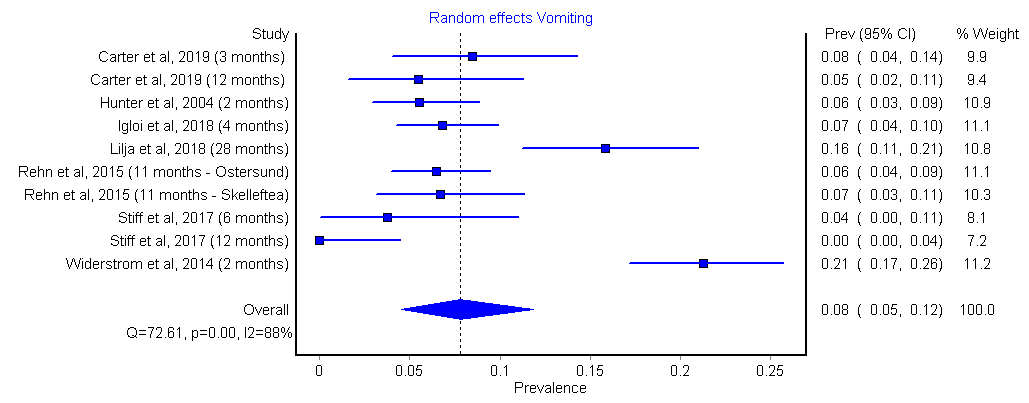


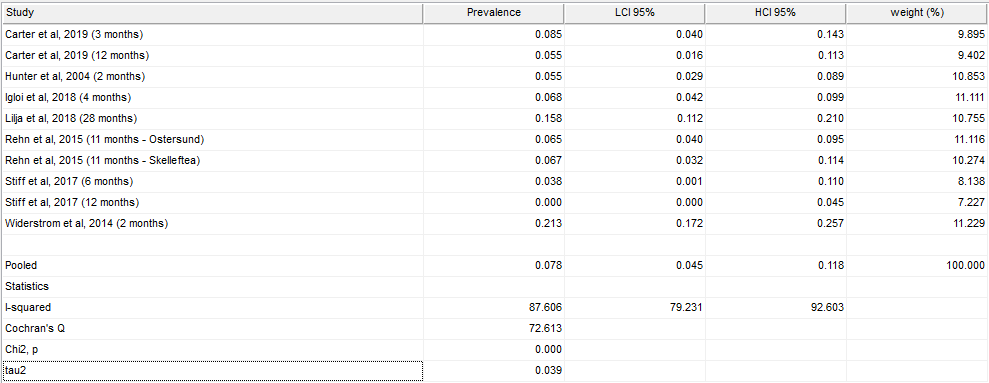


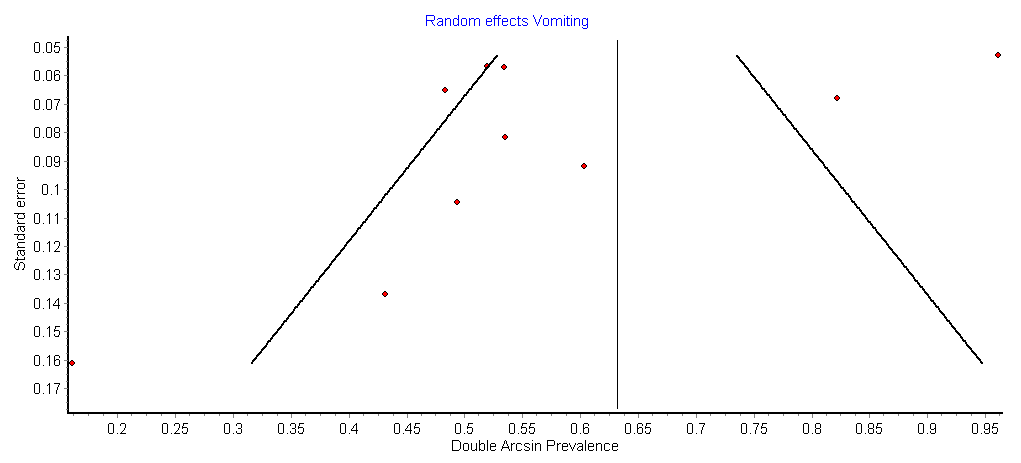


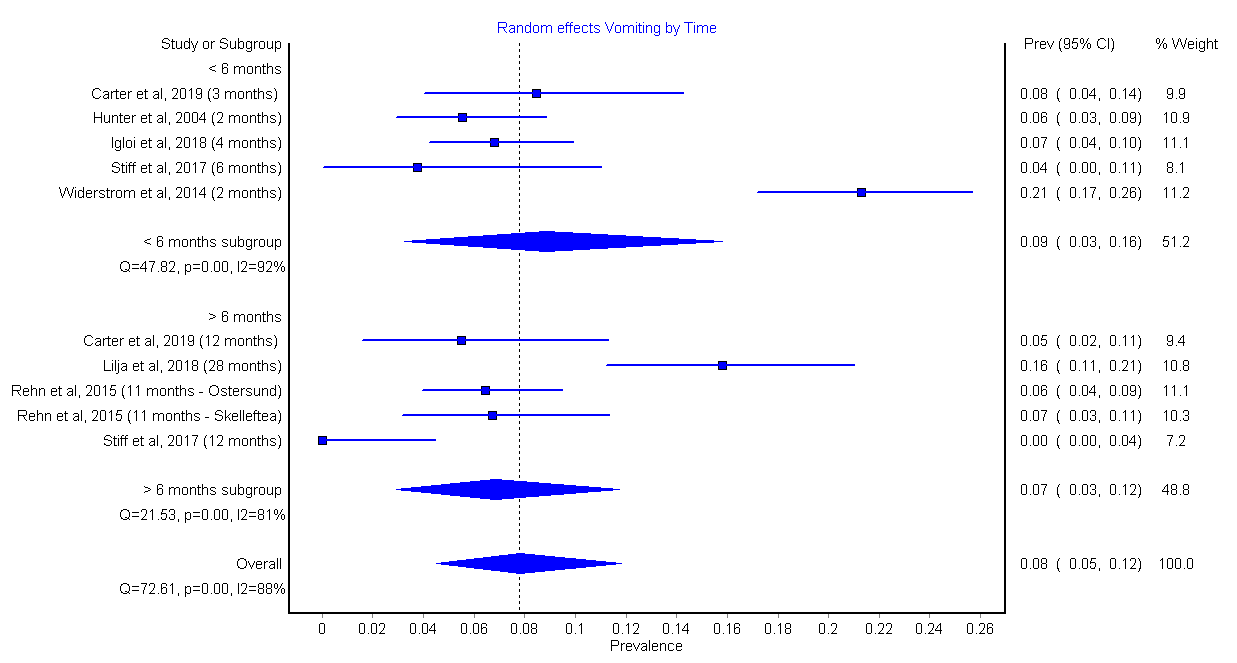


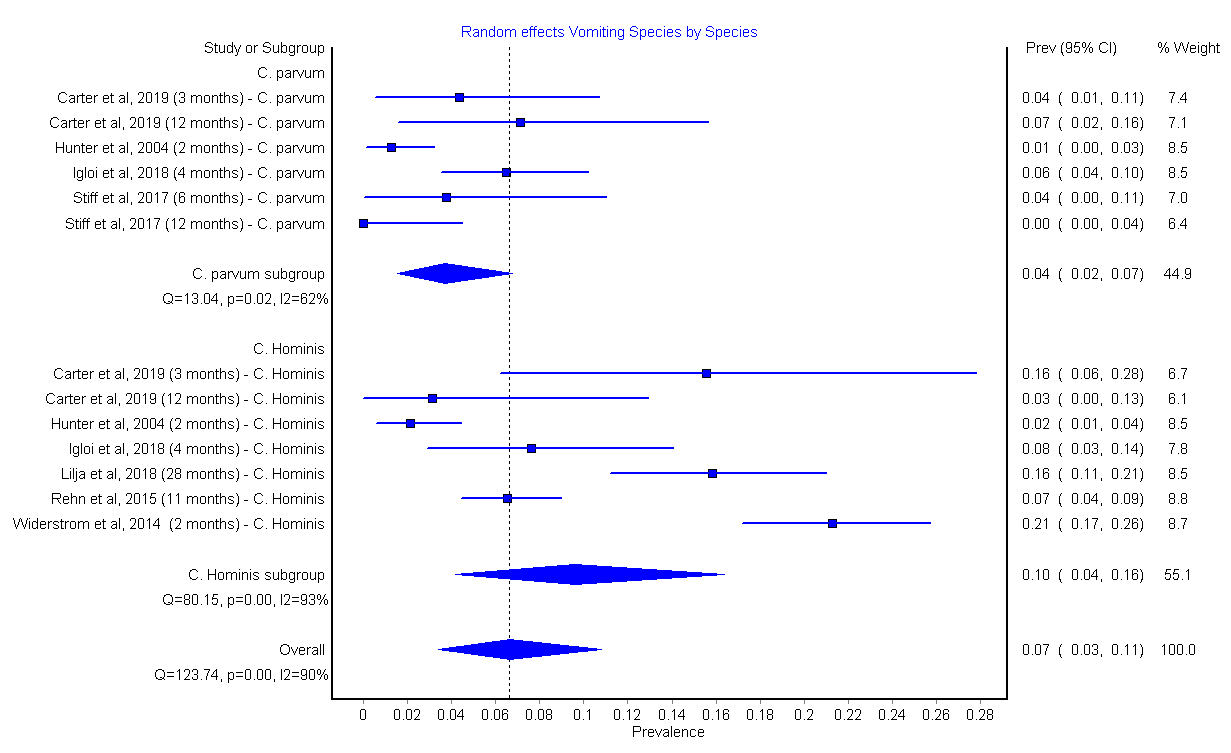


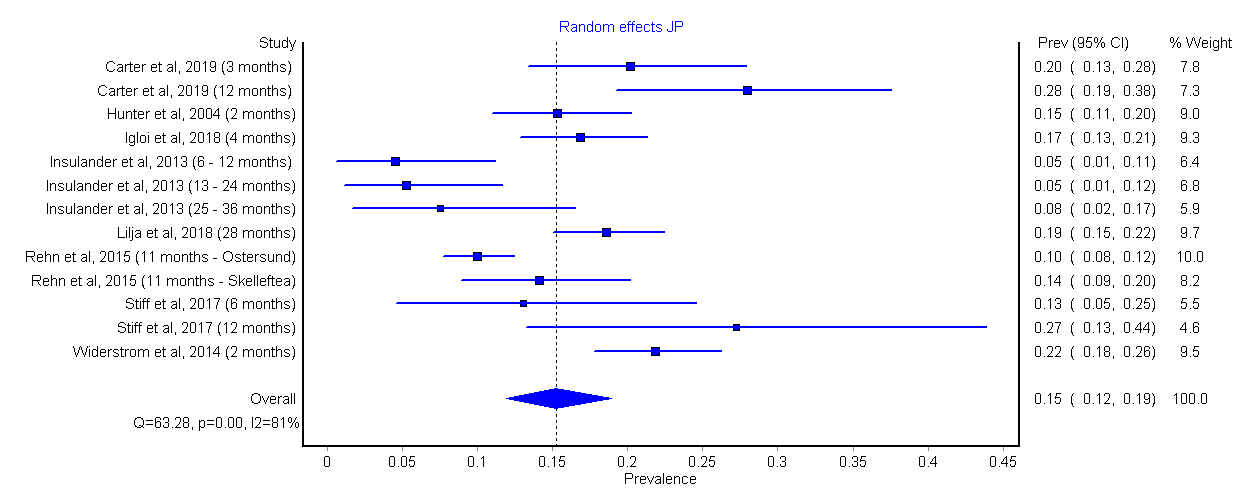


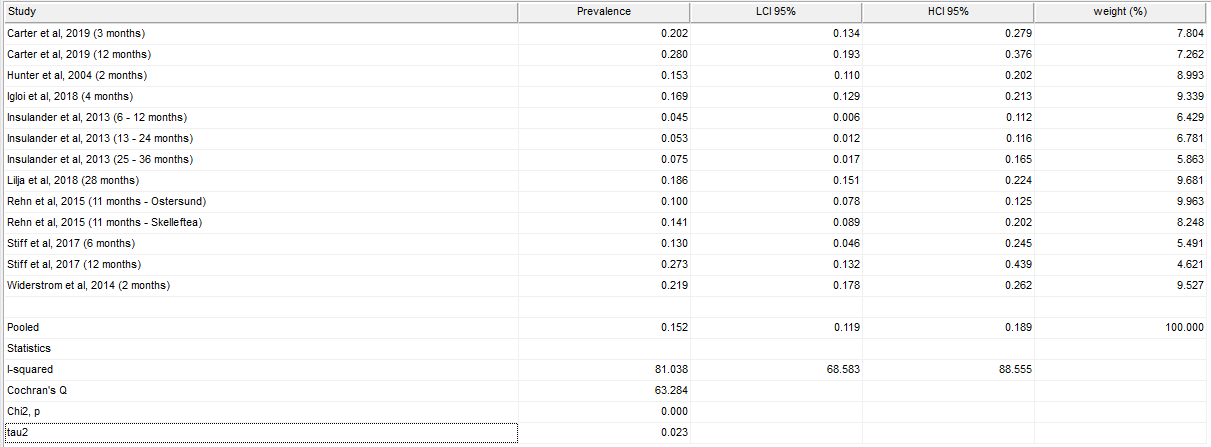


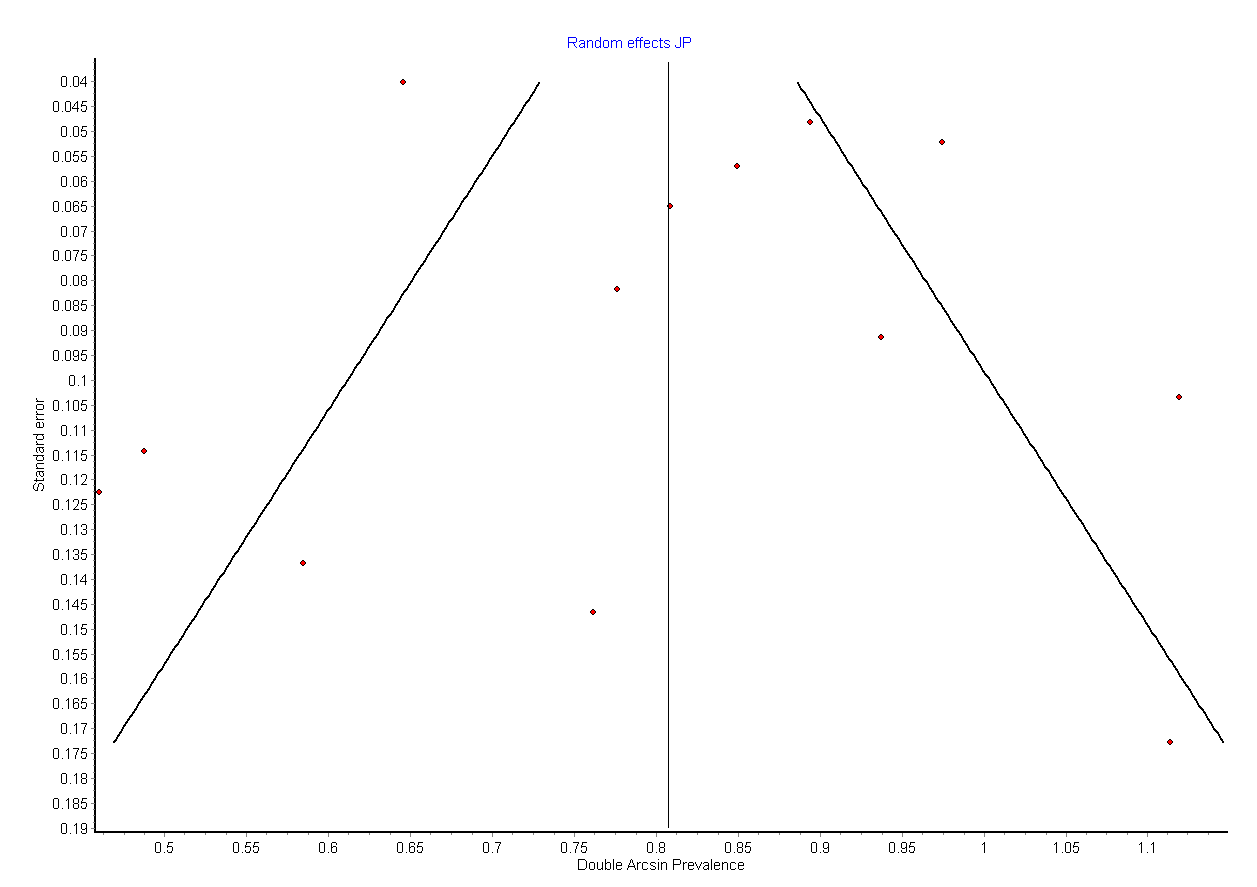


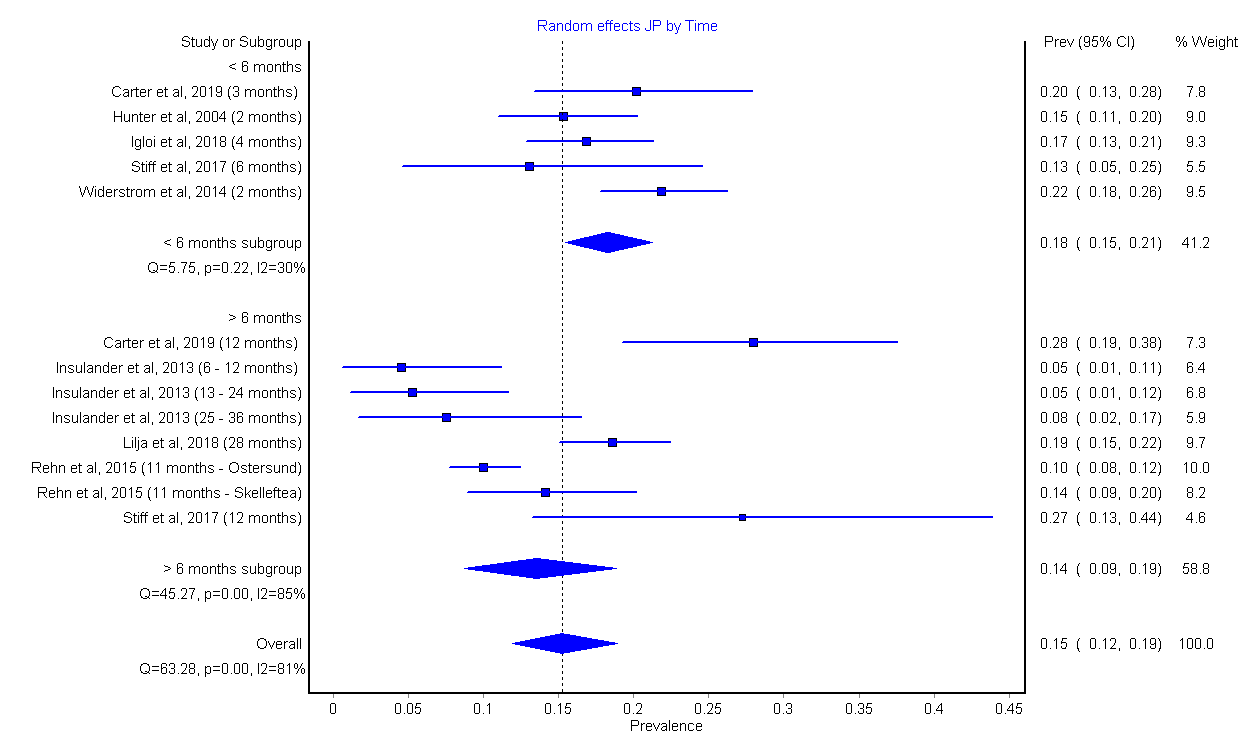


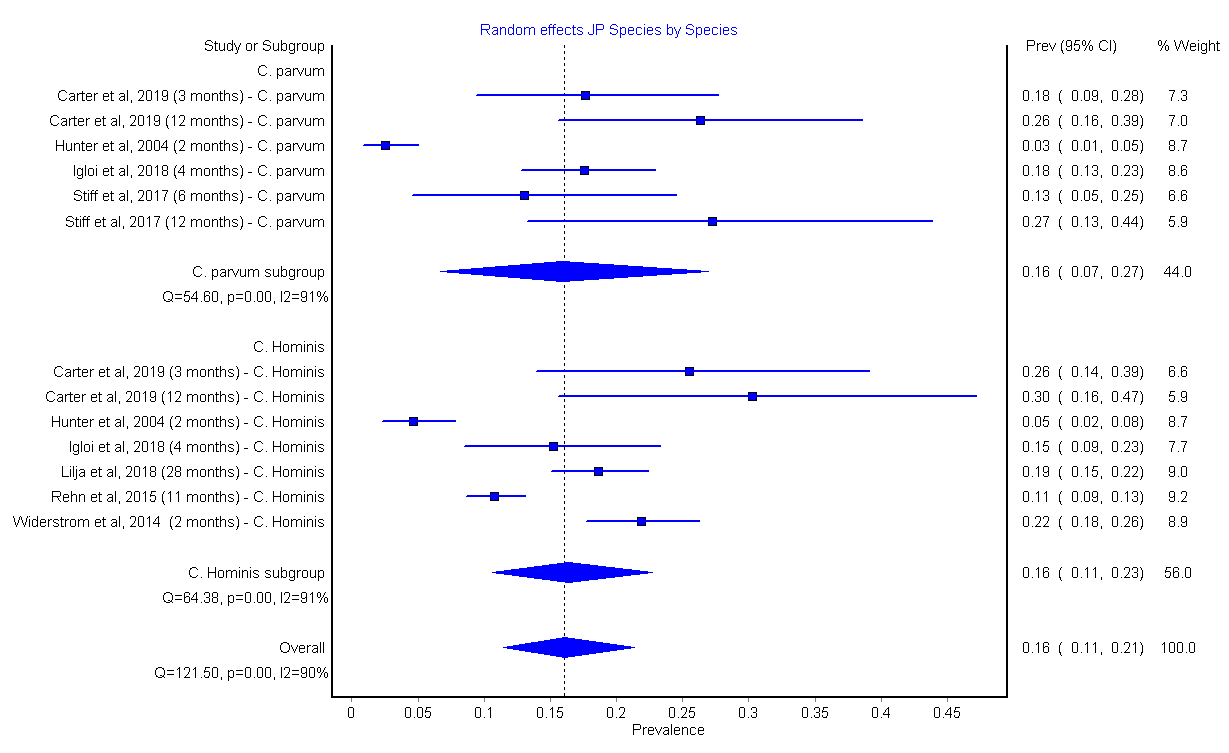


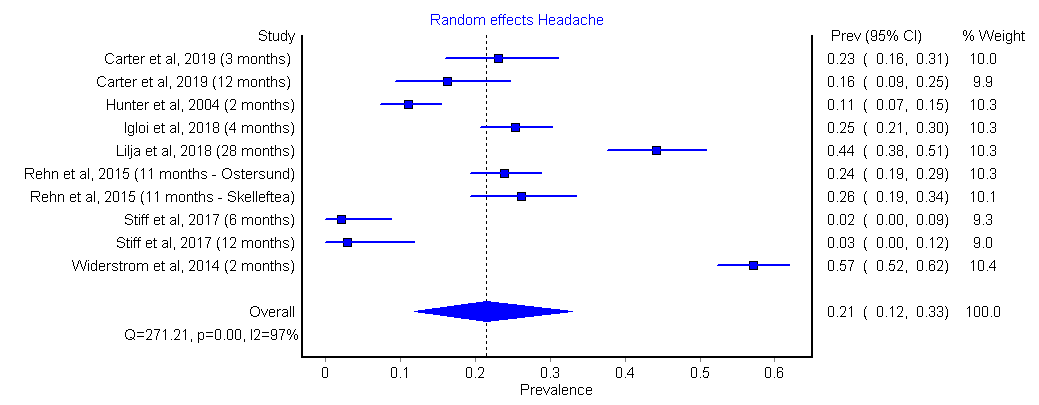


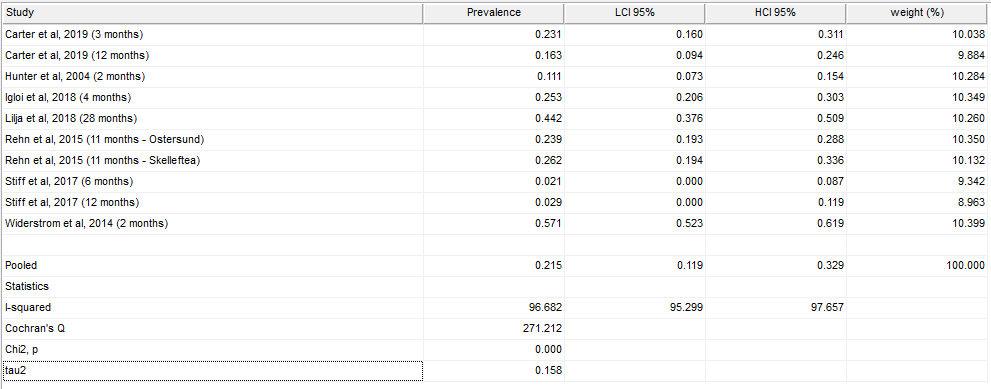


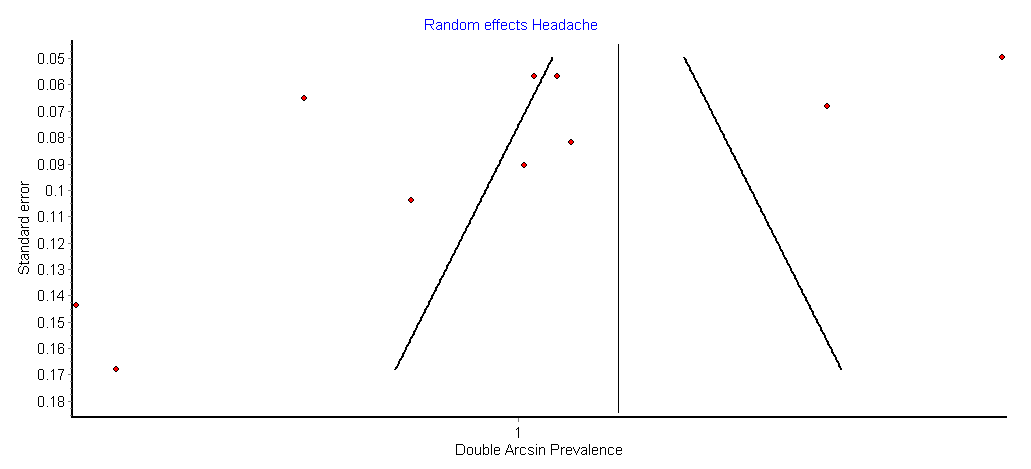


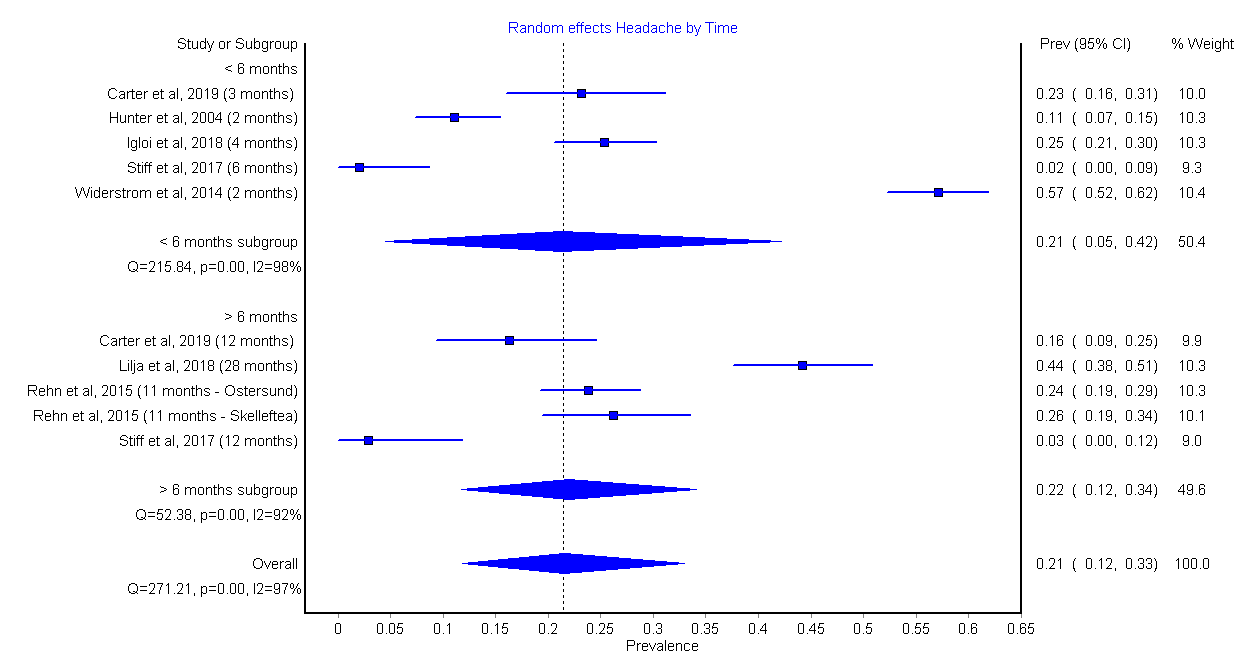


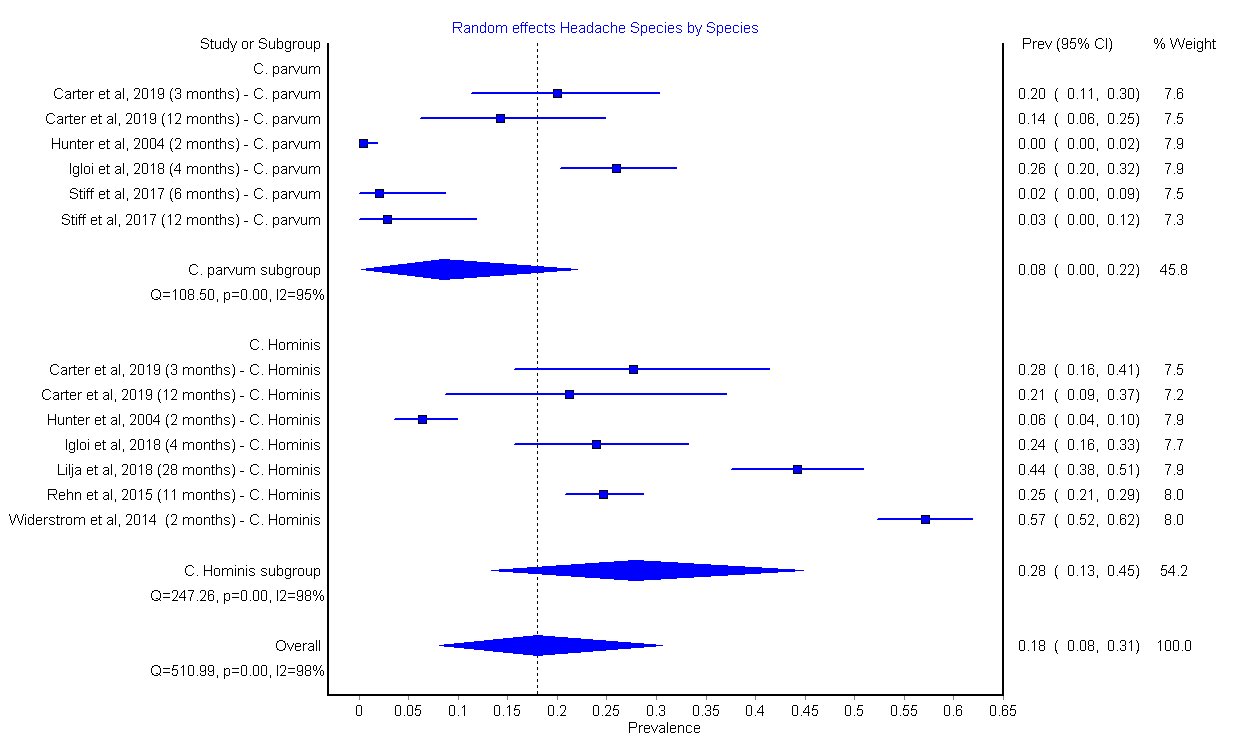


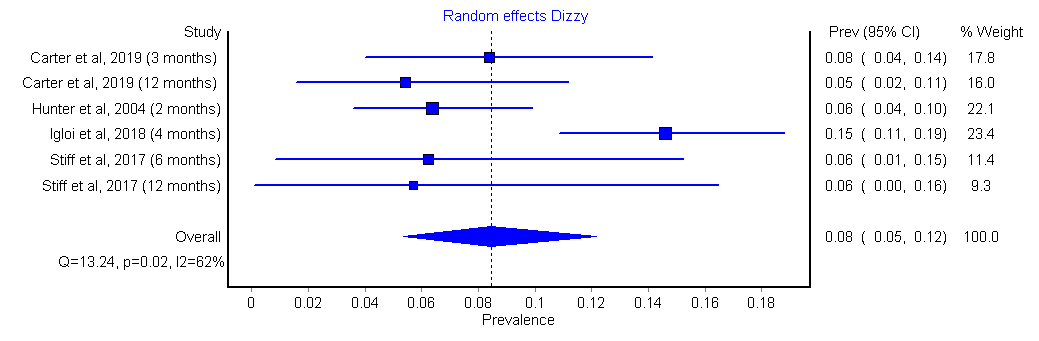


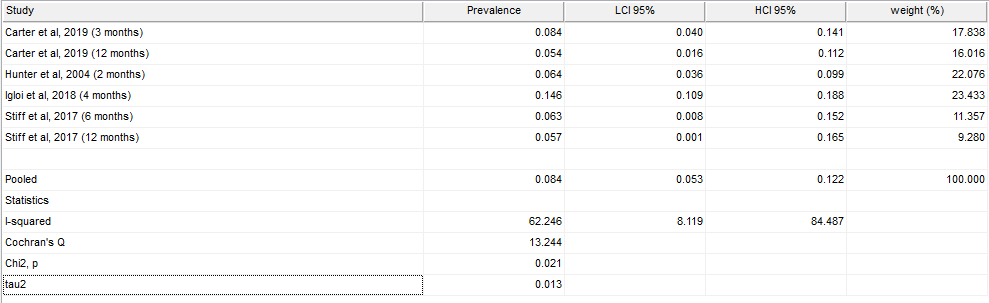


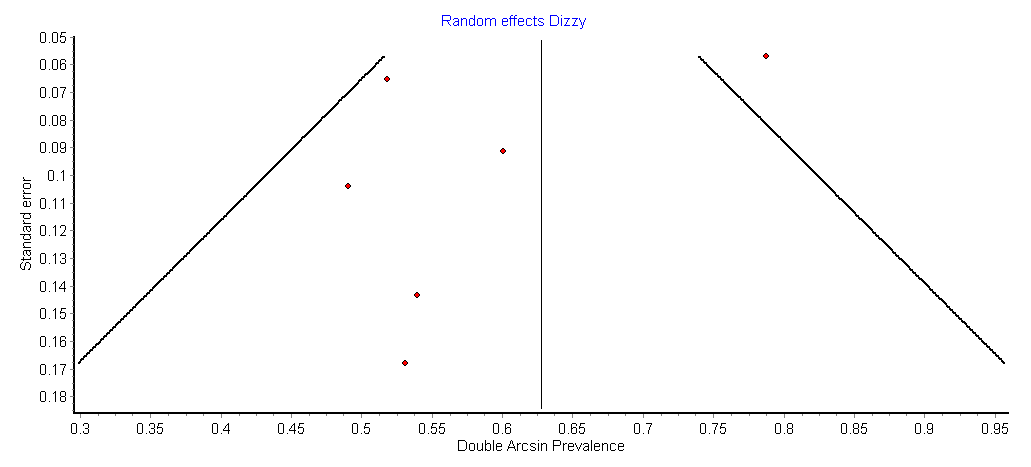


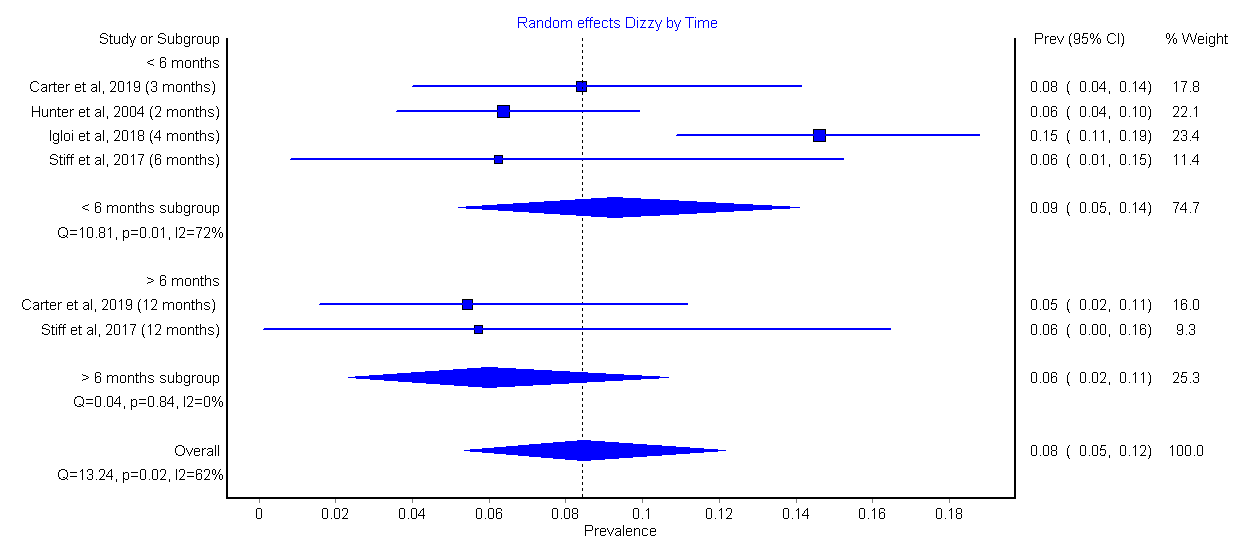


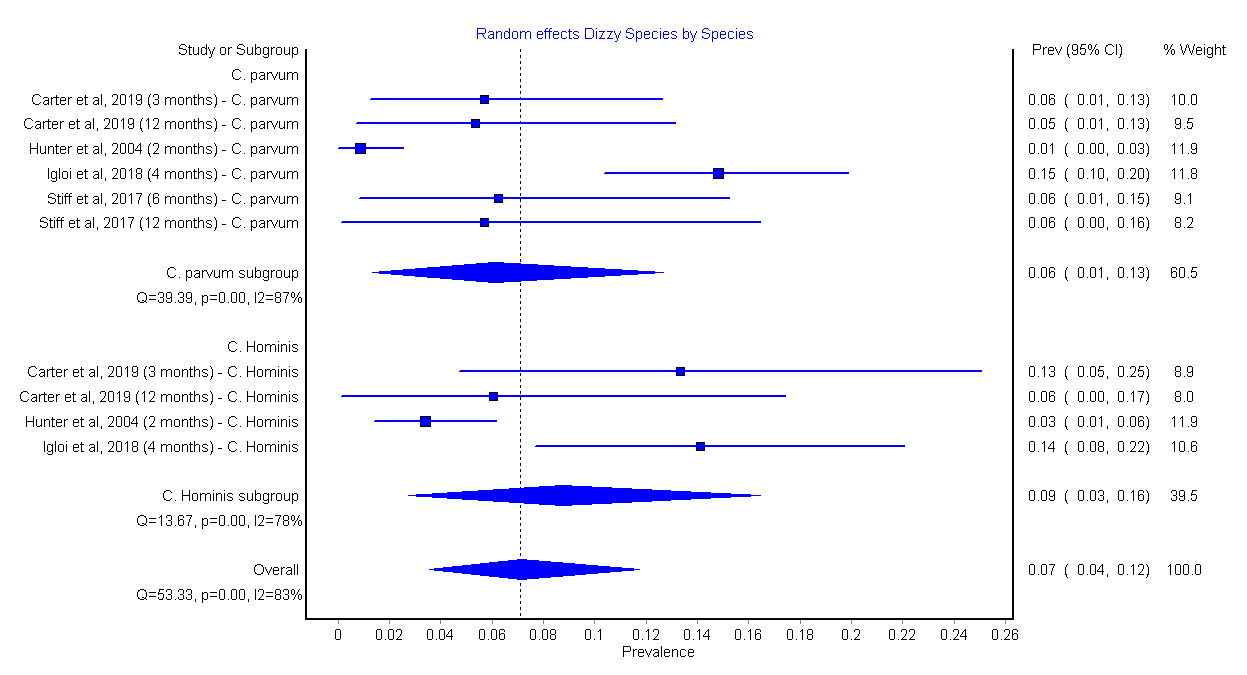


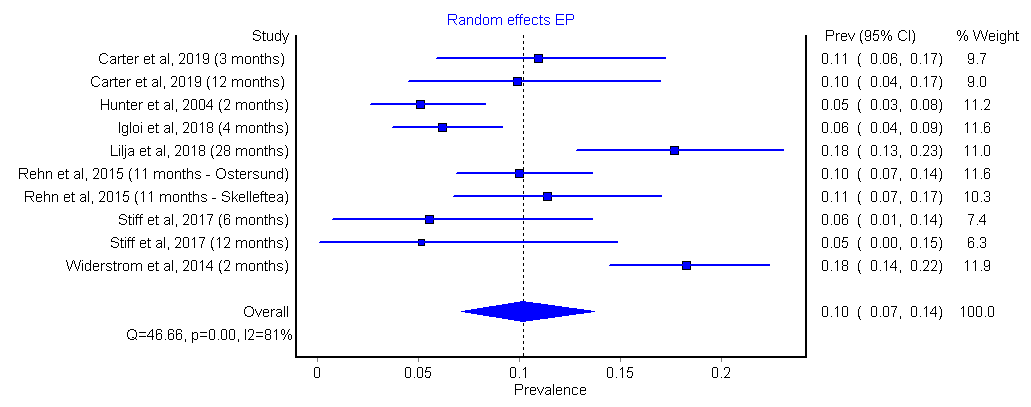


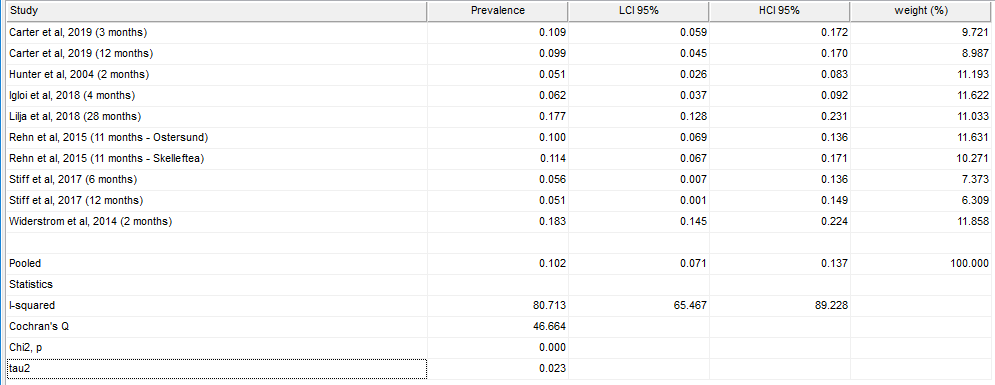


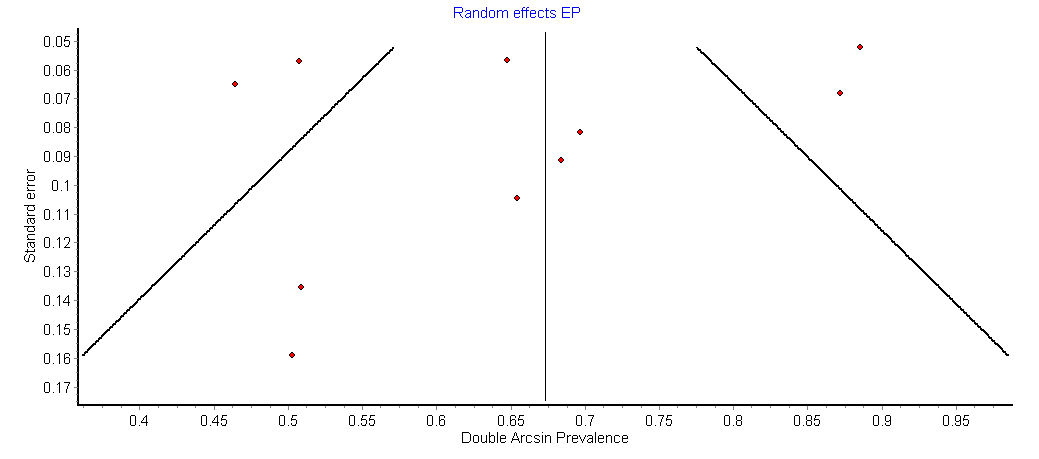


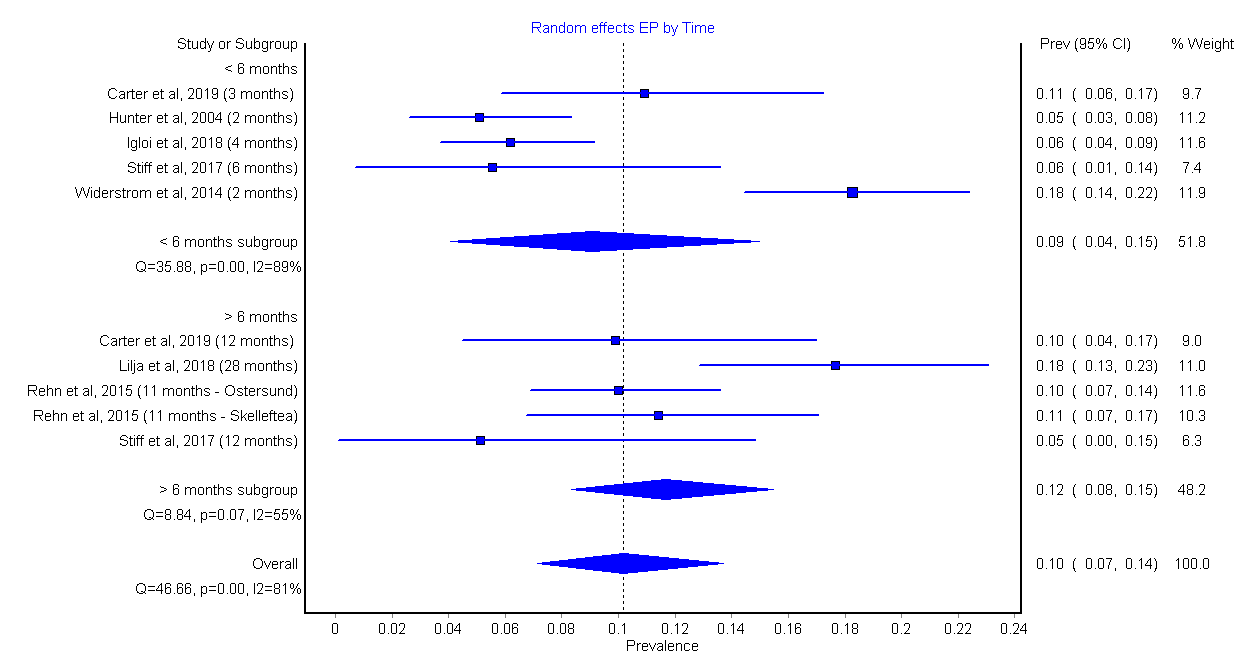


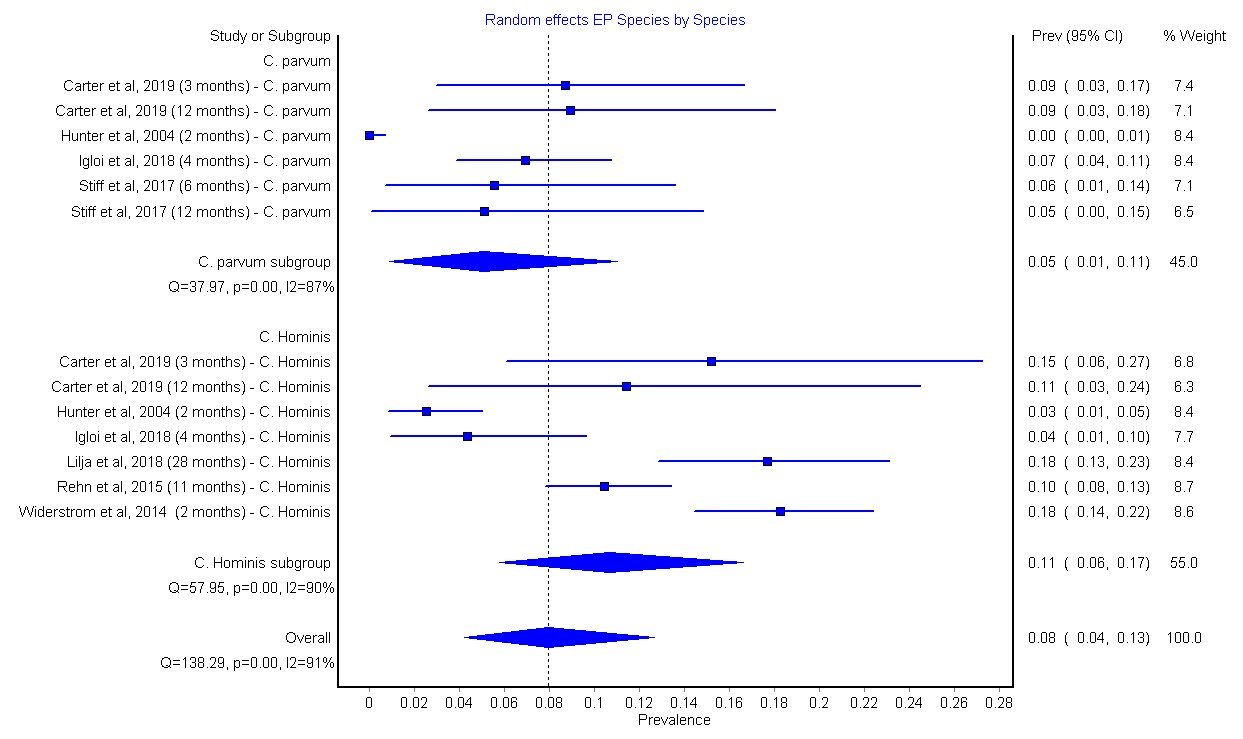


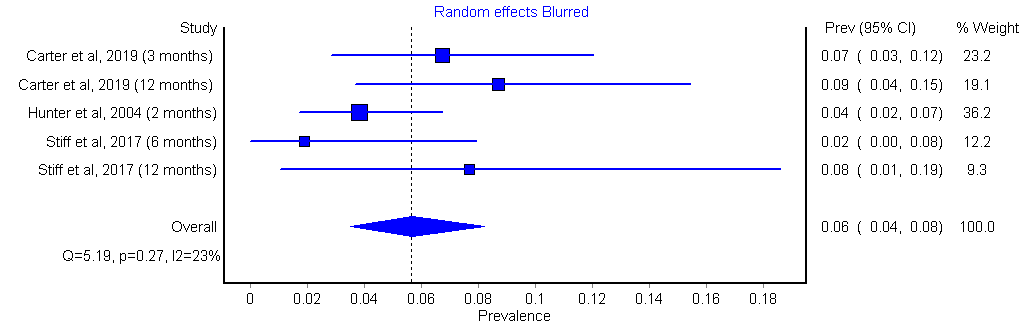


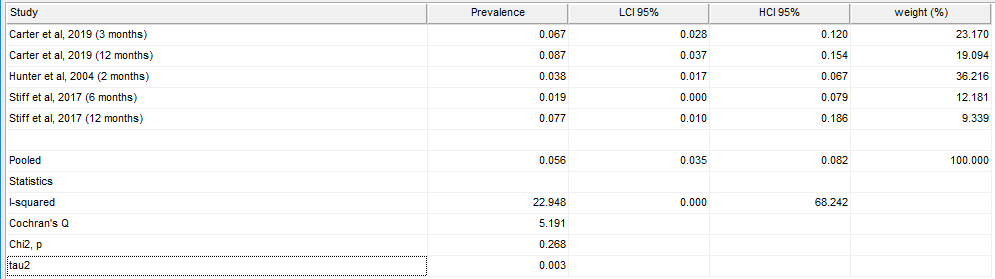


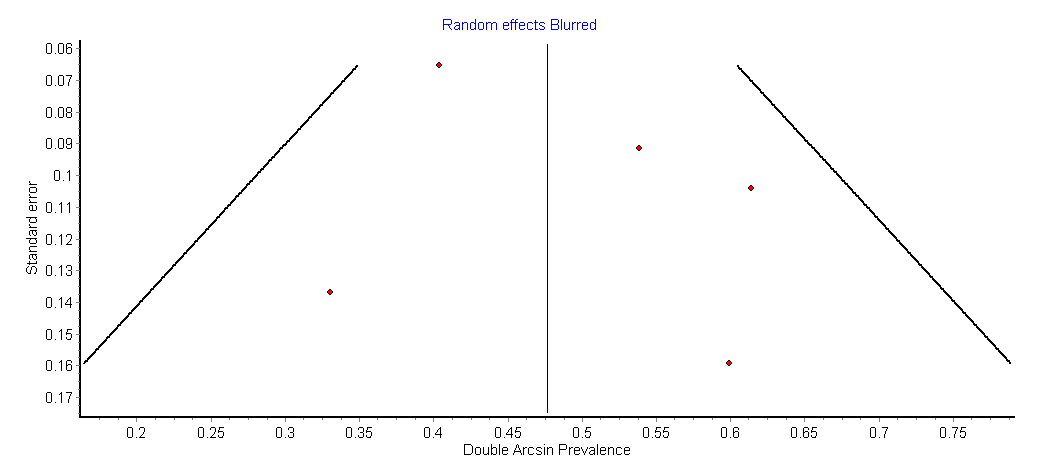


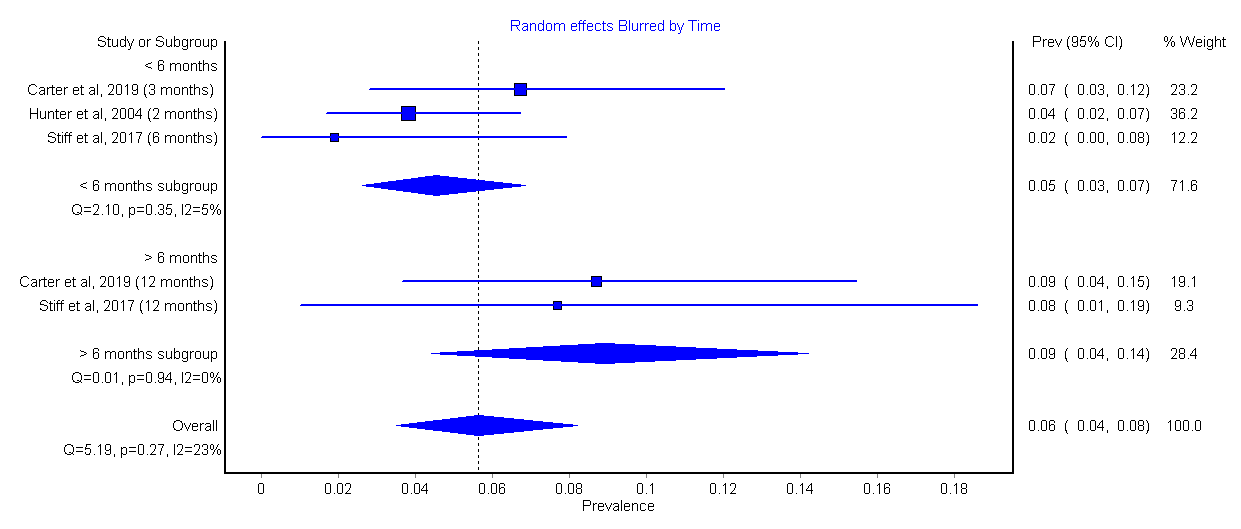


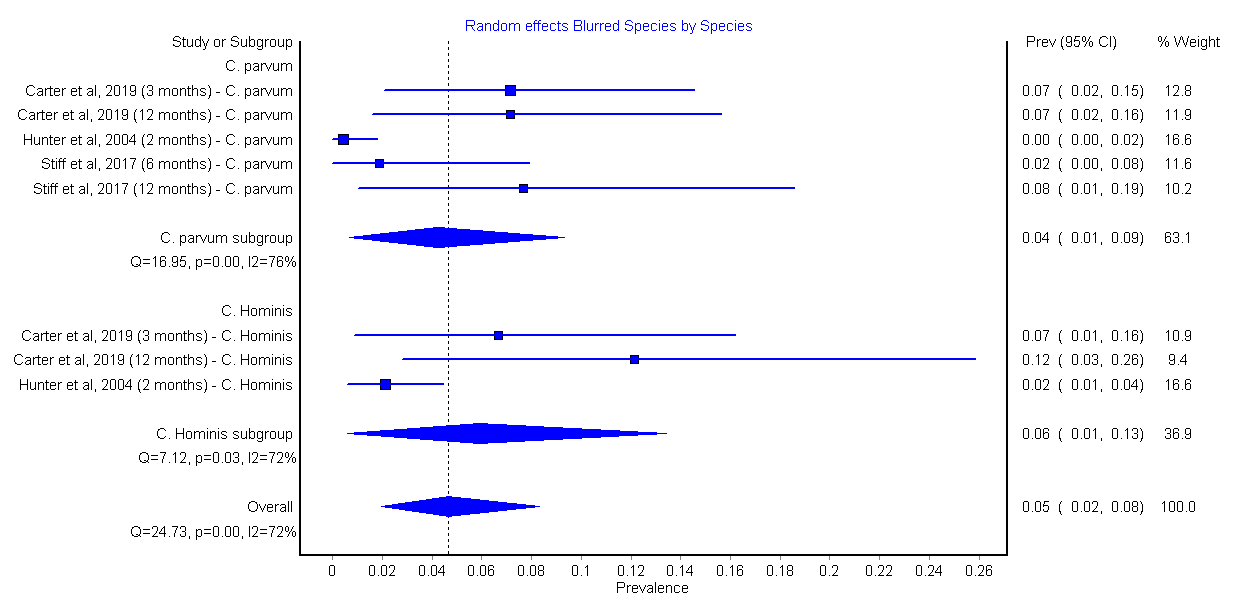


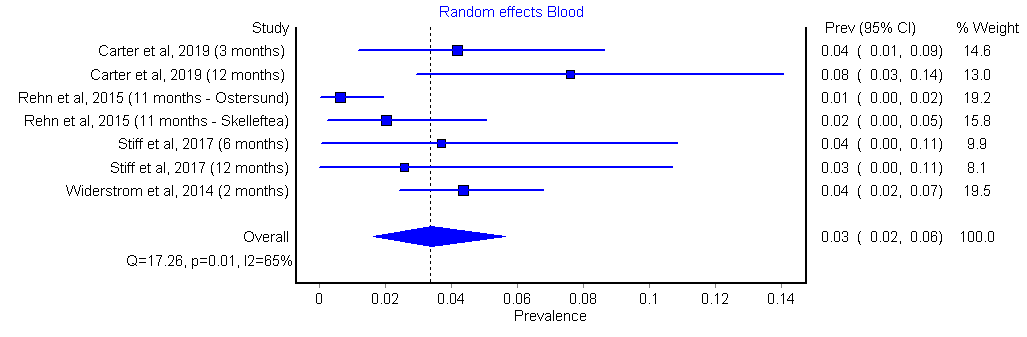


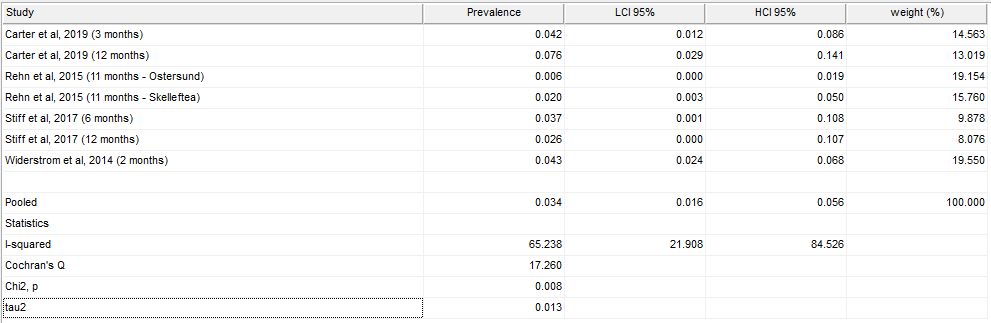


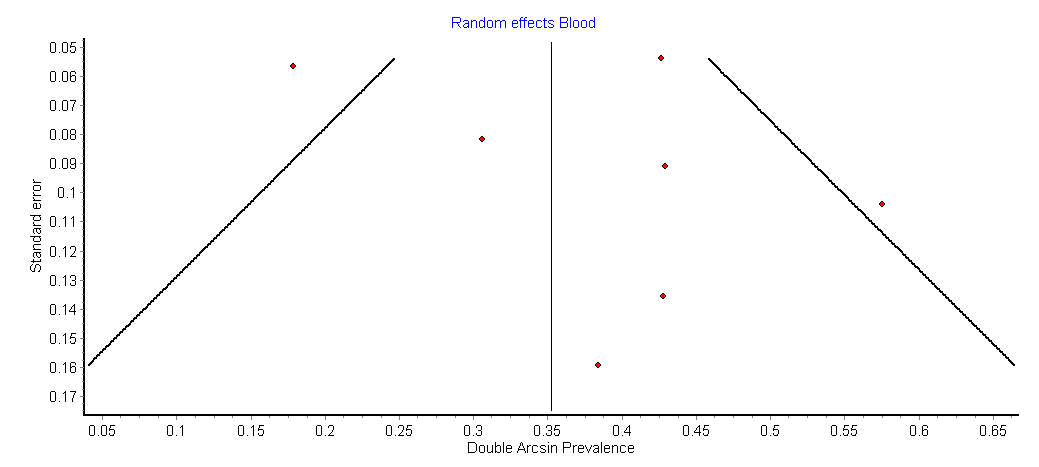


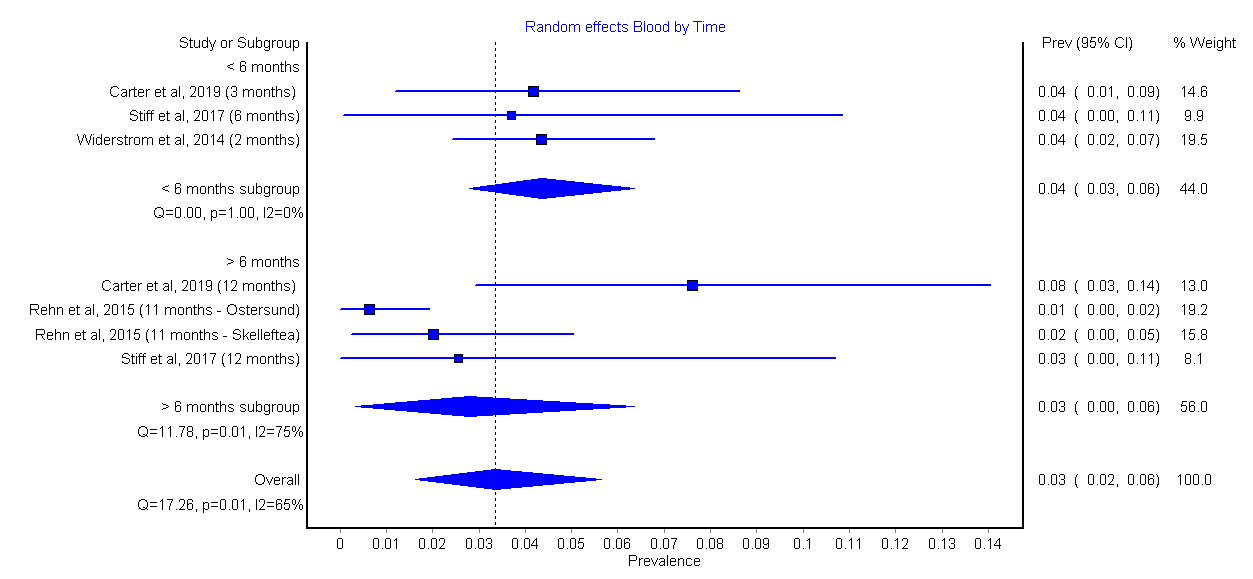


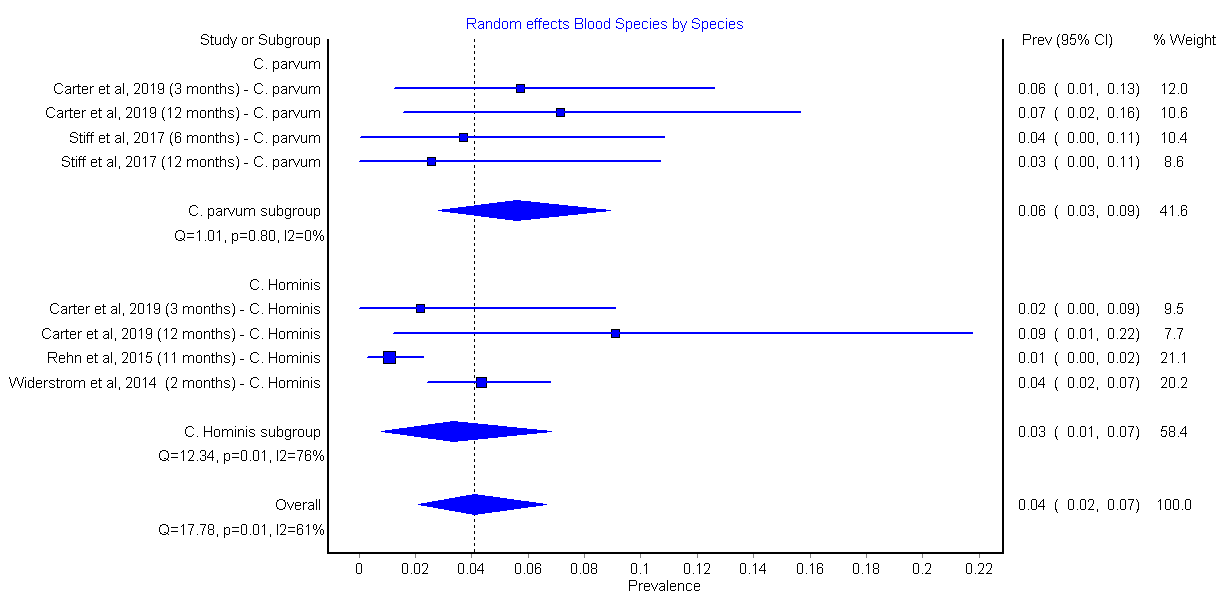


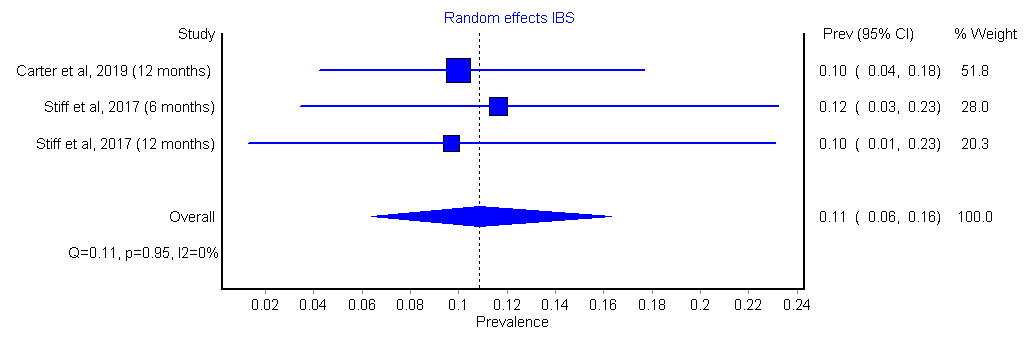


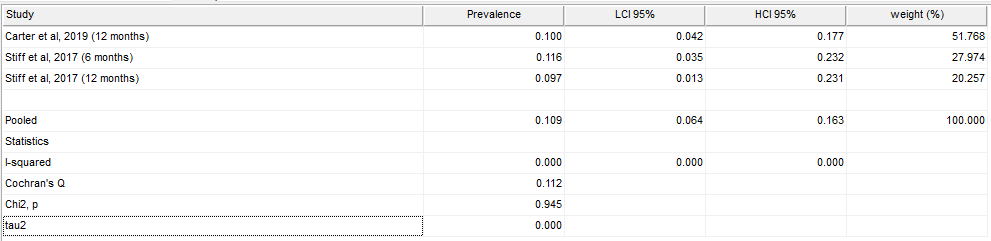


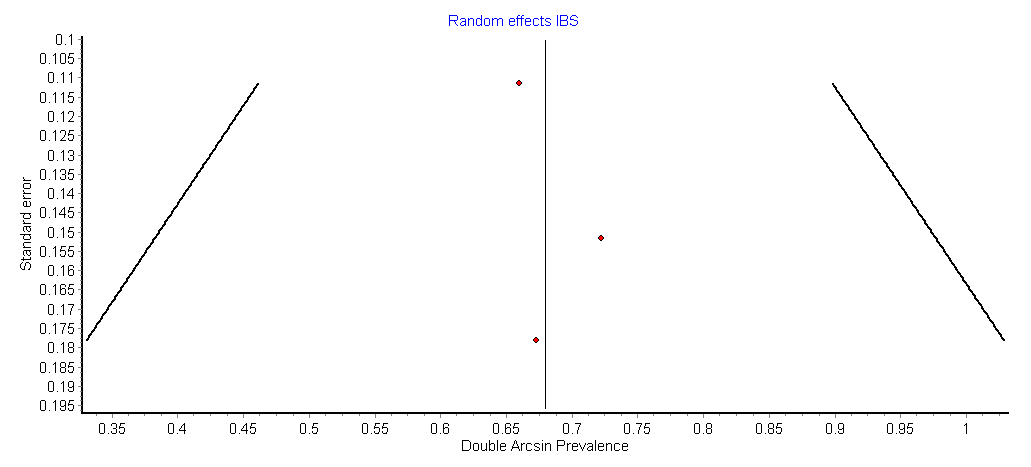


Risk

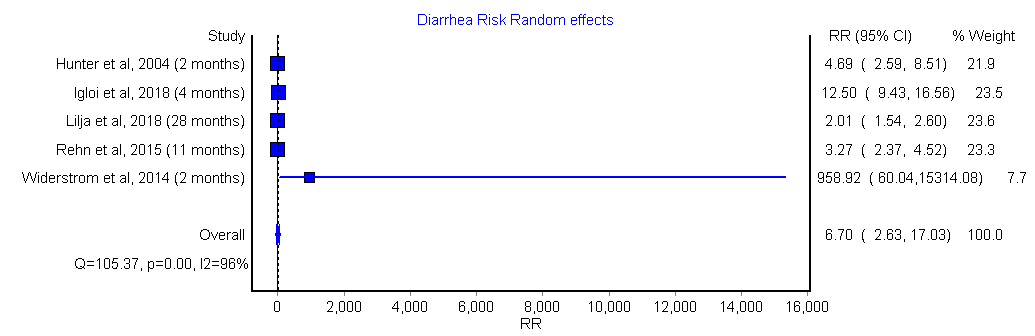


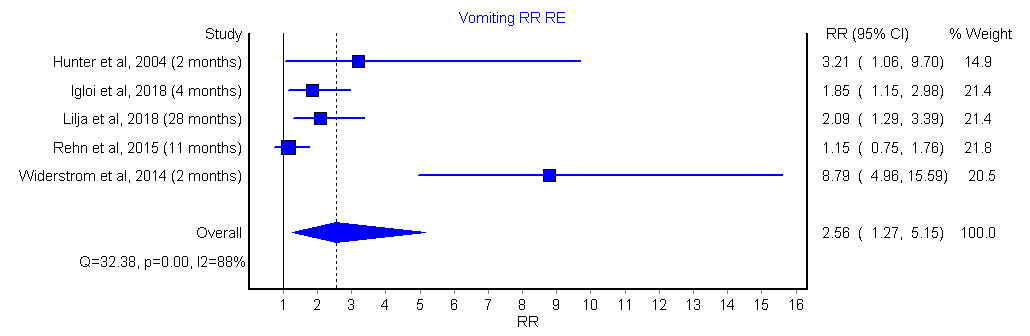


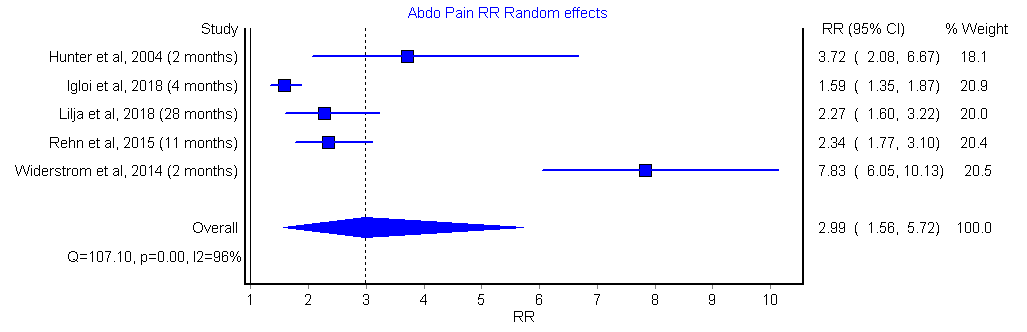


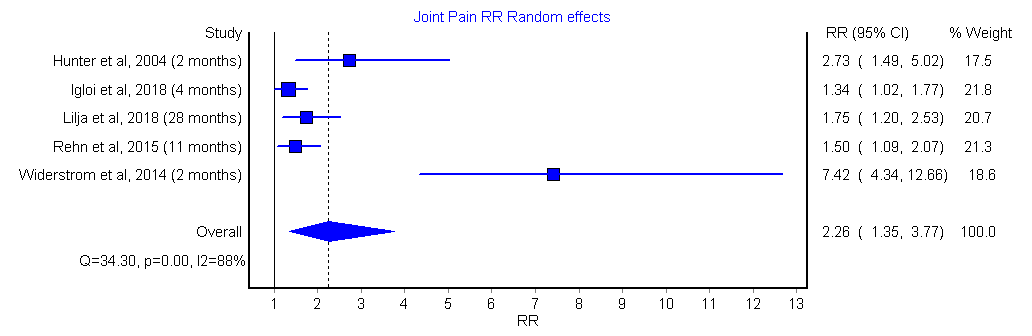


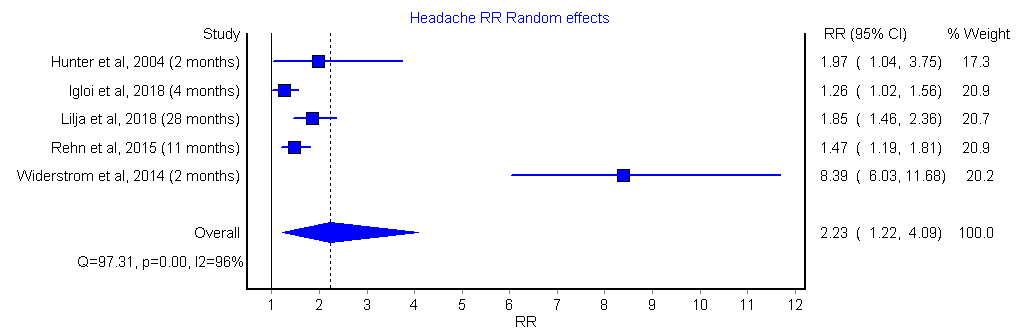


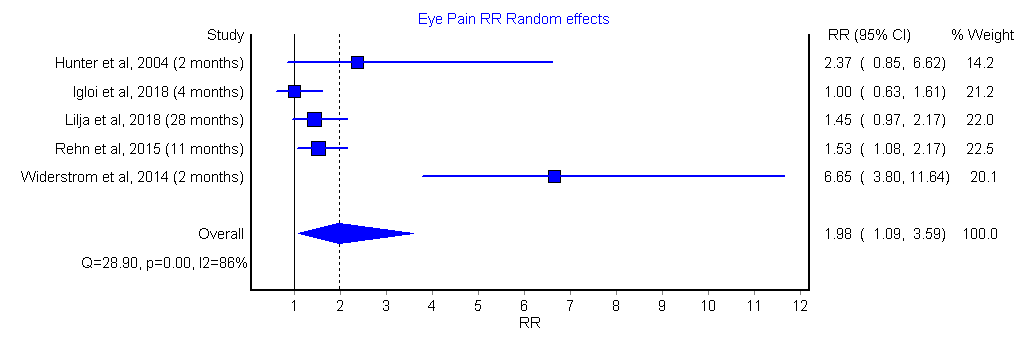


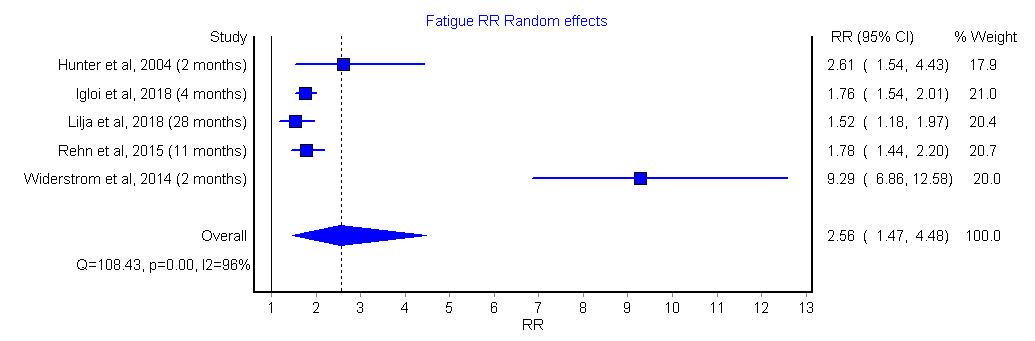


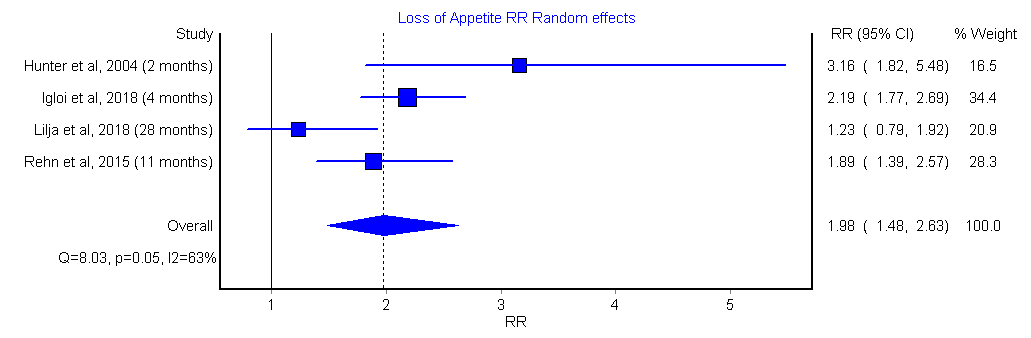


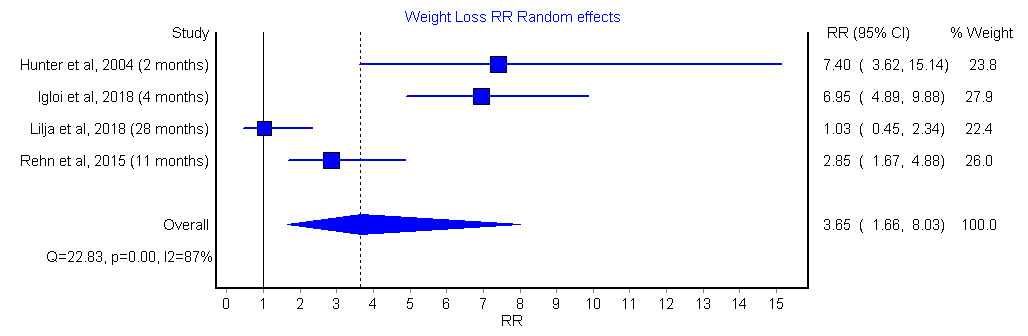

Supplement: Supplementary file 2 — Additional file 2. Data for individual sequelae. [file 13071_2020_4308_MOESM2_ESM.docx]
